# The understanding of complex syntax in children with Down syndrome

**DOI:** 10.12688/wellcomeopenres.14861.2

**Published:** 2019-02-28

**Authors:** Pauline Frizelle, Paul A. Thompson, Mihaela Duta, Dorothy V. M. Bishop

**Affiliations:** 1 Department of Speech and Hearing Sciences, University College Cork, Cork, Munster, Ireland; 2 Department of Experimental Psychology, University of Oxford, Oxford, Oxon, UK

**Keywords:** Complex syntax, Down syndrome, children, receptive language, relative clause, complement clause, adverbial clause

## Abstract

**Background:** Down syndrome (DS) is associated with poor language skills that seem disproportionate to general nonverbal ability, but the nature and causes of this deficit are unclear. We assessed how individuals with DS understand complex linguistic constructions, and considered how cognitive ability and memory and impact the ability of those with DS to process these sentence types.

**Methods:** There were three groups participating in the study: children with DS (n = 33) and two control groups composed of children with cognitive impairment of unknown aetiology (CI) (n = 32) and children with typical development (n = 33). The three groups did not differ on raw scores on a test of non-verbal cognitive ability. Using a newly devised animation task, we examined how well individuals with DS (n = 33) could understand relative clauses, complement clauses and adverbial clauses compared to children with CI and typically developing controls. Participants also completed the Test for the Reception of Grammar-2, three measures of memory (forward and backward digit recall, visuo-spatial memory) and a hearing screen.

**Results:** Results indicated that (1) with the exception of intransitive subject relative clauses, children with DS performed at floor on all other complex sentences, (2) they performed at a significantly lower level than both control groups, and (3) DS status accounted for a significant proportion of the variance over and above memory skills.

**Conclusions:** Our findings suggest that children with DS have a disproportionate difficulty understanding complex sentences compared to two control groups matched on mental age. Furthermore, their understanding of syntax is not completely explained by poor cognitive or memory skills, rather it appears to be a specific deficit that may distinguish children with DS from other neurodevelopmental disorders.

## Introduction

Down syndrome (DS) is the most common genetic cause of intellectual disability. A diagnosis of DS is given when an error in cell development results in an extra copy of chromosome 21, so there are 47 chromosomes rather than the usual 46. DS can also be the result of mosaicism, when only some cells include an extra copy of this chromosome, or translocation, when part of chromosome 21 attaches to another chromosome.

The majority of individuals with DS have a moderate intellectual disability ( Chapman & Hesketh, 2001); however, IQ scores can span from the severe to the average range ( Roizen, 2007). Language difficulties in children with DS are well documented, particularly those affecting vocabulary, phonology, morphology, and simple sentence structures ( Dodd & Thomspson, 2001; Eadie *et al.*, 2002; Laws & Bishop, 2003; Price *et al*., 2007). However, information regarding these children’s understanding of complex syntax is very limited. In addition, although children with DS have increased risk of a number of difficulties likely to influence their language development (involving limited cognitive ability, hearing level and memory skills) the relationship between these factors and language competence is not straightforward and has never been investigated in relation to the complex syntactic abilities of this population. We aim to address this gap in the literature.

Our previous research has shown that children's performance on language comprehension tests can be heavily influenced by task demands ( Frizelle *et al.*, 2017). Previous studies have tended to use multiple-choice tasks that have a heavy cognitive load and make demands beyond the linguistic. Given that people with DS have a cognitive impairment, we anticipated that these tasks may underestimate their comprehension abilities. For the current study, we used a novel method of assessment, designed to minimize non-linguistic demands. We hypothesised that this may reveal a greater level of syntactic understanding than when using a traditional multiple-choice format.

### Complex syntax

The term ‘complex sentence’ is used to refer to constructions that have more than one clause, linked in specific ways. This can be done through co-ordination (using connectors such as *and* or *but*) or subordination, where there is a main clause in which an element is embedded or expanded into a subordinate clause. Subordination is of particular interest as it allows for the expression of thoughts that involve hierarchical relationships between ideas, rather than just chaining them together. There are three distinct types of subordinate clause; complement clauses, adverbial clauses and relative clauses, and all three are the focus of the current paper. Examples of each clause type are given in Table 3. Complement clauses are the earliest developing form of complex sentence ( Diessel, 2004) and are often used with mental state verbs such as *know* and *think.* In a complement clause, the embedded sentence serves as one of the arguments of the verb in the matrix clause ( Quirk *et al.*, 1985). The complement clause can therefore be the subject, object or indirect object of the main verb. In this paper we are concerned with complements that serve as the object of the main clause. In adverbial constructions the two clauses are linked semantically, most commonly using temporal (e.g. *when*) or causal (e.g. *because*) connectives. Finally, a relative clause serves to post-modify the noun in the main clause. They are usually defined according to (a) the sentential position of the modified noun phrase and (b) the role of the relativized noun phrase in the embedded clause. In this study, in keeping with children’s early production of relative clauses (see Diessel, 2004), we focused on relatives that modify the main clause object. In addition, we included relative clauses, where the relativized noun phrase realizes a range of syntactic roles, such as subject, object, oblique and indirect object.

### Language characteristics of children with DS

The characteristic profile of language abilities in those with DS suggests that receptive language is typically better than expressive language ( Chapman *et al.*, 2002; Laws & Bishop, 2003) and that vocabulary is stronger than syntax. The latter is evident in both receptive and expressive modalities ( Abbeduto *et al.*, 2003; Berglund *et al.*, 2001; Chapman *et al.*, 1991).

Much of the work in relation to syntax has focussed on expressive language and primarily on spontaneous language production. Given the marked differences between children with DS and their age-matched peer group, it is customary to compare their language profiles with that of younger, typically developing (TD) children. This makes it possible to see whether language development is merely following a typical, but markedly delayed, course, or whether there is a distinctive profile with strengths and weaknesses in specific aspects of language. Individuals with DS have been reported to produce fewer complex noun phrases, verb phrases, sentence structures, questions and negations than TD individuals of a similar non-verbal age ( Price *et al.*, 2008). A limited production of passives has also been reported ( Bridges & Smith, 1984; Fowler, 1990; Ring & Clahsen, 2005). In relation to complex syntax specifically, Thordardottir *et al.* (2002) analysed 12-minute narrative samples from 24 adolescents with DS (mean age 16.5 years) and a control group of younger TD children matched on mean length of utterance (MLU). Co-ordinated sentences, clausal complements and relative clauses were all noted in the narrative samples, with no significant differences between the groups in either the proportion or the diversity of complex sentences used. However, Thordartottir and colleagues did highlight the degree of variability in the group with DS.

More recently, Christodoulou & Grohmann (2018) reported on the comprehension of syntactically complex subjunctives (e.g. The cat wants to dance) in 30 Greek Cypriot bilectal adolescents with DS. Using an act out priming task followed by a picture selection task they found a high rate of comprehension accuracy in their DS participants and posit that their results contradict previous suggestions of an overall syntactic impairment in people with DS. However, a close look at the distractor items suggests that the participants could respond correctly by understanding key words in the sentence and did not need to understand the complex syntax i.e. for the sentence above, from an array of 4 pictures there was only one image depicting a cat dancing, therefore if the participant understands the words ‘cat’ and ‘dance’ they are likely to choose the correct picture.

Other studies have reported deficits in syntactic comprehension in individuals with DS, (see Fortunato-Tavares *et al.*, 2015; Michael *et al.*, 2012; Perovic, 2006; Ring & Clahsen, 2005) however, the range of structures investigated is narrow and complex syntax has been given little or no attention. When complex syntax is involved, it has been in the context of standardized measures, in which different syntactic structures (both simple and complex) are grouped together and a composite score is reported (for example using The Test for Auditory Comprehension of Language-Revised (TACL-R) ( Carrow-Woolfolk, 1985) or the Test for the Reception of Grammar (TROG-2) ( Bishop, 2003). It is, accordingly, not possible to tease out the potential contribution of the complex constructions included in these tests to the scores achieved.

In addition, we have found that children's performance on language comprehension tests can be heavily influenced by the specific demands of the assessment method employed ( Frizelle *et al.*, 2017). The format used in the TACL-R and TROG-2 is the traditional multiple-choice sentence picture-matching presentation, where the goal is to select from an array the picture that matches a spoken sentence. The other images are distractors that represent alternative interpretations of the sentence and the child is required to rule them out in order to respond correctly. These competing interpretations are presented so that only children with a deep understanding of the construction will chose the correct item. However, this format is likely to lead to children failing for reasons other than a lack of linguistic knowledge. In particular, it can disadvantage children (such as those with DS) who are inattentive and impulsive, and those who do not appreciate the need to scan the array carefully to choose between similar-looking items. We developed a new test (TECS-E: Test of Complex Syntax-Electronic) that was designed to minimise such demands by using a format where the child sees a specific animation and has to judge whether it matches a spoken sentence. Because this is in effect a two-choice test, it is necessary to give at least eight items per structure to distinguish chance performance from understanding. Using this approach we found that TD children as young as 3;06 years showed understanding of some complex constructions that they had found difficult when tested using the more traditional multiple choice picture-pointing approach (see Frizelle *et al*., 2018a). Of course, no method is completely free of task demands or item-specific influences on performance, but our experience of the TECS-E with young children raised the possibility that traditional approaches to assessing comprehension may underestimate understanding in children with DS.

### Receptive language and cognition in DS

Although it is tempting to consider cognitive ability as a core factor in explaining receptive language differences between those with DS and other groups, the literature is not consistent in this regard, particularly in relation to vocabulary. Several studies suggest that the receptive vocabulary of those with DS is in keeping with that of cognitively matched children with typical development, ( Chapman *et al.*, 1991; Laws & Bishop, 2003; Miller, 1995) while other studies suggest a lower performance from those with DS ( Caselli *et al.*, 2008; Hick *et al.*, 2005; Price *et al.*, 2007). The literature regarding syntactic comprehension appears to be more homogenous with the majority of studies showing that those with DS have a lower than expected understanding of syntax relative to their non-verbal cognitive skills ( Abbeduto *et al.*, 2003; Chapman *et al.*, 1991; Joffe & Varlokosta, 2009; Laws & Bishop, 2003; Price *et al.*, 2007; Rosin *et al.*, 1988). However, it is important to note that most of these studies have used the same assessment measures, with a significant focus on morphology and simple syntax and few embedded sentences. Some studies have compared those with DS to mental-age-matched TD controls, while others have matched cognitive ability with other cognitively impaired groups such as those with Williams syndrome, Fragile X syndrome and those with specific language impairment. The aim of these comparisons is to see whether there is a distinctive profile specific to those with DS relative to other groups who have a language and or cognitive impairment. While previous reports appear mixed and are somewhat dependant on the comparison group under scrutiny, existing literature suggests that children with DS perform at a similar level to those with Williams syndrome and specific language impairment. A summary of the findings comparing those with DS with other groups, on their understanding of syntax is shown in Table 1. 

**Table 1.** Understanding of syntax in children with Down syndrome relative to mental age matched controls. The grammatical morphemes subtest measures inflectional and derivational morphology. The Elaborated Sentences subtest measures simple and multiclause sentences – e.g., active or passive voice, direct or indirect objects.

Study	Sample	Language measures	Performance relative to mental age matched controls
Abbeduto *et al.* (2003)	DS (n = 25) Fragile X (n = 19) TD (n = 24)	TACL-R subtests Grammatical morphemes Elaborated sentences	DS vs Fragile X = significantly poorer DS vs TD = slightly poorer
Chapman *et al.* (1991)	DS (n = 48) TD (n = 48)	TACL-R subtests Grammatical morphemes Elaborated sentences	DS vs TD = similar
Chapman (2006)	DS (n = 20) CI (n = 16) (Unknown aetiology)	TACL-R subtests Grammatical morphemes Elaborated sentences	DS vs CI (Unknown aetiology) = poorer
Finestack *et al.* (2013)	DS (n = 24) Fragile X (n = 22) TD (n = 27)	TROG-2	DS vs TD = poorer DS v’s Fragile X = similar
Joffe & Varlokosta (2009)	DS (n = 10) WS (n = 10) TD (n = 10)	TROG-2 TAPS	DS vs WS = similar DS vs TD = poorer
Laws & Bishop (2003)	DS (n = 19) SLI (n = 19) TD (n = 19)	TROG-2	DS vs SLI = similar DS vs TD = poorer
Price *et al.* (2007).	DS (n = 45) all boys TD (n = 45)	TACL-R subtests Grammatical morphemes Elaborated sentences	DS vs TD = poorer
Rosin *et al.* (1988)		Miller-Yoder Language Comprehension Test	DS vs TD = poorer
Pennington *et al.* (2003)	DS = 28 TD = 28	TROG	DS vs TD = poorer

WS, Williams Syndrome; TACL-R, Test for Auditory Comprehension of Language-Revised ( Carrow-Woolfolk, 1985); TROG-2, Test for the Reception of Grammar, 2 ^nd^ edition ( Bishop, 2003); TAPS, Test of Active and Passive Sentences ( van der Lely, 1996).

### Memory characteristics of children with DS

Individuals with DS show particular difficulties with verbal short-term or working memory tasks ( Jarrold & Baddeley, 2010; Jarrold *et al.*, 2002; Laws, 2002) even when compared to other groups with cognitive delay, who do not have DS ( Bower & Hayes, 1994; Chapman, 2006; Laws, 2004). In contrast, their visual memory skills are often superior to, or at least in keeping with these groups, ( Bower & Hayes, 1994; Chapman, 2006; Rowe *et al.*, 2006) suggesting that their memory deficits are specific to language.

Laws (1998); Laws (2004) reported a strong correlation between verbal short-term memory and a reduced mean length of utterance, as well as language comprehension difficulties. A strong relationship between memory and syntax has also emerged. Chapman *et al.* (2002) took a number of measures, at four time intervals over a 6-year period, from 31 individuals with DS between the ages of 5 and 20 years. They reported that along with chronological age, both verbal and visual working memory, were significant predictors of syntactic comprehension ability. In addition, Chapman & Hesketh (2001) reported these factors to be key predictors of expressive syntax at the onset of their study. The connection between memory ability and syntactic difficulties in those with DS is also evident in work by Michael *et al.* (2012). Michael and colleagues took a number of memory measures from individuals with DS, and a TD group matched on vocabulary, including digit span, word span, a spatial memory task and a sentence repetition task. Both groups performed similarly on all measures, with the exception of the sentence repetition task. They suggested that when compared to digit and word span, the syntactic processing load of a sentence was particularly difficult for the individuals with DS to parse and recall.

Because the current study focuses particularly on complex sentences, and these constructions require the parsing of different clauses over a more lengthy time span ( Martin & McElree, 2009; Marton *et al.*, 2006), we might predict that short term and working memory would be particularly relevant to the ability of someone with DS to understand them. However, to our knowledge, this has never been investigated in relation to this population.

### Current study

In sum, given the limited scope of previous research on comprehension, in terms of both methods and linguistic structures, we are uninformed about how individuals with DS process and understand specific complex structures as well as how cognitive ability, memory and hearing level impact the ability of those with DS to deal with these sentence types.

Our first aim was to investigate how well individuals with DS can understand complex structures such as relative clauses, complement clauses and adverbial clauses. Based on findings that individuals with DS produced relative and complement clauses in their narrative samples ( Thordardottir *et al.*, 2002), we hypothesised that many of those with DS would be able to understand these constructions, although, on the basis of prior literature, we anticipated considerable performance variation. We compared strengths or weaknesses seen in those with DS to two other groups: (a) those with intellectual disability but of unknown aetiology (matched to those with DS on non-verbal mental age), and (b) a group of TD younger children at the same non-verbal mental age. This allowed us to identify whether those with DS have a characteristic syntactic profile relative to the other two groups. Based on previous findings (such as those reported by Abbeduto *et al.*, 2003; Chapman, 2006; Laws & Bishop, 2004) we hypothesised that those with DS would perform similarly to those with an intellectual disability of unknown origin but more poorly than the TD group matched on non-verbal ability. Based on data from TECS-E with TD children aged from 3;06 to 5 years (see Frizelle *et al.*, 2018a) we anticipated an order of difficulty within each family of constructions (relative, complement and adverbial). Within the five types of relative clause we expected children to have the least difficulty with intransitive subject relatives, with other relative clause types being of a similar level of difficulty. Within adverbial clauses we expected causal adverbials to be the least demanding, followed by those that are temporal, with conditional adverbials causing the greatest difficulty. Finally, we anticipated that sentences using the verb *pretend* would be the least difficult complement clause items and that those using the cognitive state verb *think* would be the most difficult to understand. We based this expectation on previous (unpublished) data collected from young typically children between 3;06 and 4;11 years.

We also examined how children performed on TECS-E relative to a standardized test of grammar using the multiple-choice format. In the standardized measure (TROG-2; Bishop, 2003) children must show an understanding of the syntactically simple constructions before they progress on to those that are more complex. Therefore, by applying the discontinue rule, if a given number of items are failed, children will not be tested on complex sentences. Here, in order to compare test administrations between TECS-E and TROG-2, we always administered block S (relative clauses) from TROG-2 at the end of the test, even if the stopping criterion was reached. This block of four items uses relative clauses attached to a main clause object, two of which are similar in construction to those used in the TECS-E (albeit with some lexical differences—a noun rather that a pronoun in the head noun position: *The girl chases the dog that is jumping*) and two of which incorporate prepositional phrases ( *The cup that is on the box is red*).

Finally we considered how far comprehension difficulties in those with DS were associated with cognitive ability, verbal short-term or working memory abilities and hearing thresholds, and whether these associations differed according to the assessment format used. We predicted correlations of comprehension scores with all three variables, though performance on particular clause types would differ, as discussed above. Given the additional cognitive load involved in a multiple-choice format, we hypothesised that children’s performance on this task would correlate more highly than TECS-E with overall cognitive and memory abilities.

Our pre-registered hypotheses https://osf.io/5ntvc/ were as follows:

1) Individuals with DS will be able to understand a range of the complex sentences tested, although we expect considerable individual variation.
2) Those with DS will perform more poorly overall than TD controls but at a similar level to those with cognitive impairment of unknown origin.
3) Children will have greater difficulty understanding comparable constructions on the multiple-choice test than on the animation task.
4) Cognitive ability, verbal memory, working memory and hearing level will predict performance in the DS group.
5) Cognitive, verbal and working memory abilities will account for more variance on the multiple-choice than on the animation task.

## Methods

### Power analysis

For our main hypothesis, the best estimate of effect size came from prior studies that compared those with Down syndrome to TD controls on composite measures of syntactic comprehension. We calculated the average effect size for the difference between those with Down syndrome and TD controls matched on nonverbal mental age. We made the assumption that the effect size for the difference between those with cognitive impairment of unknown origin and those with typical development is similar in magnitude to the previous average effect. Cohen’s *d* values were calculated for each piece of metadata and then converted to *f* ^2^ for use in the sample size calculation. The conversion was done for two groups using the formula *f* ^2^ = *d* ^2^/ *2k*, where *k* is the number of groups and *d* is Cohen’s *d*. We use used *f* ^2^ as our measure of effect size as this corresponded to our method of analysis, linear multiple regression.

The median effect size from prior literature was 0.19. This was entered into G*power software (F test, linear multiple regression: Fixed model, R ^2^ increase (a priori; see Faul *et al.*, 2007). The sample size required with two tested predictors (intellectual level and Down syndrome status) at 90% power and alpha = 0.05 was 70, giving an estimate of 23 participants per group.

We had anticipated that our subsequent analyses would incorporate the additional predictors of memory and hearing level, giving a total of five predictors. With five predictors we estimated a total sample of 93 (at 90% power and alpha = 0.05). However, we also expected to be able to drop one predictor, depending on the results of our first analysis. If there was no group effect, the group term could be dropped, and if the Down syndrome group differed from the other two groups, then the group comparison could be coded in one variable. The effect number of predictors would therefore be four, with a required total sample size of 86. Accordingly we aimed for a sample of 30 participants per group.

### Participants

A total of 47 participants with DS were recruited to the study. The study was conducted between November 2017 and May 2018, in the Republic of Ireland, where the prevalence of DS is one in every 546 live births ( Johnson *et al.*, 1996). Parents/guardians confirmed the DS diagnosis. Children were recruited through local parent support groups, postings on social media and through organizations representing people with DS. Of those recruited, 14 were subsequently excluded; 4 children were non-verbal and therefore did not meet the expressive criterion below; two were unable to attempt the experimental task; 1 had a significant hearing loss and 2 were in the severe to profound range of intellectual disability. This resulted in 33 children with DS participating in the study. With the exception of 5 children with DS who attended special schools, all others attended mainstream schools. To avoid floor effects only those with a non-verbal mental age of 3;06 years and above on the Leiter International Performance Scale 3 ^rd^ Edition (Leiter-3) ( Roid *et al.*, 2013) were included and children were required to be capable of producing 3-word utterances at a minimum. The sample size was calculated using a power analysis from a hierarchical linear regression analysis, with an expected effect size of 0.19 (see below for justification).

For the comparison groups, 32 children with cognitive impairment of unknown aetiology (CI) and 33 TD children were also recruited into the study. Those with typical development were recruited from mainstream schools and preschools, and those with CI were recruited from both special and mainstream schools. Both groups were matched on cognitive ability to those with DS, using the Leiter-3 ( Roid *et al.*, 2013) (F (2, 95) = 2.077, *p* < 0.131). TD children were included on the basis that they had never been referred for speech and language therapy, had typical language abilities (based on teacher and parental reports), and had no known neurological or hearing difficulties. All groups spoke English as their first language and the language of the home, and both control groups underwent hearing-screening tests across the same frequency range as those with DS. The descriptives for each group are given in Table 2.

**Table 2.** Characteristics of Participants with Down syndrome (DS), cognitive impairment of unknown aetiology (CI) and typical development (TD).

Variable	DS (N = 33, 12 boys)		CI (N = 32, 17boys)		TD (N = 33, 16 boys)	
	Mean	SD	Mean	SD	Mean	SD
CA, months	115.24	15.51	119.31	16.45	77.67	9.57
NV IQ	69.24	6.72	70.66	7.15	102.18	10.13
MA, months	79.27	9.73	83.91	11.97	79.21	10.08
FD span	2.36	0.65	3.72	0.73	4.76	1.06
FD Accuracy	8.78	3.90	17.43	5.86	27.21	8.26
BD span	1.36	0.49	1.66	0.83	2.60	0.79
BD Accuracy	4.33	4.52	8.03	6.94	17.48	6.46
VS Span	2	0.97	1.97	0.78	2.90	0.93
VS Accuracy	19.03	8.06	19.06	6.74	27.33	8.78
Hearing threshold	11.94	0.36	11.94	0.35	11.90	0.29

CA, chronological age; NV IQ, non verbal IQ (as measured by the Figure Ground, Form completion, Classification and Sequential Order subtests of the Leiter-3); MA, mental age; FD, forward digit; BD, backward digit; VS, visuo-spatial.

**Table 3.** Example test sentences for each complex sentence.

Relative clause	Example sentence
Subject intransitive	He found the girl that was hiding.
Subject transitive	He pushed the girl that scored the goal.
Object	The boy picked up the cup that she broke.
Oblique	The man opened the gate she jumped over.
Indirect object	She kissed the boy she poured the juice for.
**Complement clause**	
Think	She thinks the boy’s hair is dry
Know	He knows the girl broke the chair
Pretend	The boy is pretending he ate the chocolate
Wish	The man wishes he was on the bus
**Adverbial clause**	
Before	The boy played football before he watched TV
After	The box fell after the man opened it
Because	The girl cried because the boy pushed her
If	If the boy was taller he could get the teddy

### Ethics

The Cork Teaching Hospitals Ethics Committee granted ethical approval for the study (ECM 4-07/10/14). Informed written consent was obtained from the parents/guardians of all participants. Each participant also completed an assent form.

### Procedure

Assessments were administered in a quiet room at the preschool, school or special school that each participant attended. The assessments were completed in three sessions and included the following:

***Leiter-3.*** This is a non-verbal test of cognitive ability involving four core subtests: figure ground, form completion, classification and sequential order. Figure ground is a visual interference task, which involves the identification of images embedded within a picture stimulus that gradually increases in complexity. Form completion assesses the ability to recognize the ‘whole object’ from a randomly displayed array of its fragmented parts. Classification is a sorting task that involves categorizing objects or geometric shapes (such as coloured circles) and sequential order requires the child to understand the relationship between shapes/blocks in order to find the missing elements at the end or in the middle of a series. The test is designed to require no language for administration.

***Memory assessments.*** These included versions of the digit recall and backward digit recall subtests from The Working Memory Test Battery for Children (WMTC-C) ( Pickering & Gathercole, 2001), adapted for delivery through a laptop. Digit recall involves immediately recalling a series of numbers in the order they were presented and is considered a measure of verbal short-term memory. Two measures were taken from each of the digit recall tasks: (a) the number of trials in which the participant successfully recalled all the items in their correct serial order (span); (b) the number of trials in which the participant recalled all the items in each set presented, regardless of the order (accuracy). The former is the more typical way that items are measured on span tests. However, because relatively low levels of performance were expected, in order to avoid floor effects, the latter measure was also included. Backward digit recall involves repeating a list of digits in reverse order and is regarded to be a measure of working-memory, as it requires both the storage and processing of information. To reduce the likelihood of floor effects additional practice items were given when administering this subtest. It is worth noting that the use of an accuracy measure in relation to backward digit recall makes this task more similar to a simple span task rather than one of working memory. However, one could argue that the very process of trying to recall the digits in reverse order may add a layer of executive demand.

To ensure a consistent presentation both subtests were administered with the aid of a tablet. Within each number sequence, individual numbers were highlighted on the tablet screen to indicate the pace at which they should be read aloud. The screen was visible to the administrator but not to the person completing the test. Using the touch screen the administrator inputted the numbers as the participant recalled them. If the participant changed their mind, a reset button allowed the administrator to re-enter the digits recalled. Responses were stored and scored automatically on the tablet.

Visuo-spatial memory was assessed using a version of the block recall test from the WMTB-C ( Pickering & Gathercole, 2001) adapted for tablet presentation. Participants were presented with an array of nine identical images (of a leaf) behind which there were cartoon monsters. Beginning with one and gradually increasing in number, monsters were revealed for a period of 4 seconds. The participant was required to recall the location of the monsters by touching the appropriate leaves on the screen. The programme was designed so that the number of attempts could only equal the number of target monsters. Again two measures were taken for this task: the number of trials in which the child successfully recalled the monsters in the correct order presented (span) and the number of trials in which the child recalled all the monsters in each set presented regardless of the order (accuracy). Scoring was automated and saved to a .csv file.

The memory tasks were used as positive controls in the study, to confirm that the DS group had a cognitive profile characteristic of that previously described in the literature, therefore giving results regarded as typical of this population. We expected to replicate the finding that participants with Down syndrome have poor verbal short-term memory but preserved visuo-spatial memory relative to mental-age-matched controls ( Jarrold & Baddeley, 2010).

***Hearing***. The hearing of each participant was tested using a Madsen (Micromate 304) portable screening audiometer. This testing took place in the same room used for the language assessments. Pass/fail data was collected for each ear at 25 and 45 dB. Participants were tested at 1000, 2000 and 4000 Hz. The total number of passes achieved for both ears on all tested frequencies, was calculated as the hearing status variable.

***TROG-2.*** TROG-2 ( Bishop, 2003) is a multiple choice sentence picture-matching task. Participants listened to a target word or sentence and from a choice of four, they were required to identify the corresponding picture. In the usual administration, items are presented in blocks, each focusing on a particular grammatical structure. Syntactically simple sentences are presented first followed by those that are more complex. Individuals are required to pass all four items within each block and testing is discontinued when the individual fails five consecutive blocks. For the purposes of this study, we always administered block S from TROG-2 at the end of the test, even when the stopping criterion was reached. This block of four items uses relative clauses attached to a main clause object, similar to the sentence types used in the sentence verification task described below.

***TECS-E complex syntax comprehension task.*** This is a newly devised sentence verification task using animations, which was presented on a Microsoft Surface Pro 4 tablet computer with a 12.3” (2736 x 1824 pixel) touch screen display. The tablet was placed on a table in front of each participant. Participants were shown 114 test animations in total, each with an accompanying auditory test sentence. All test sentences were pre-recorded by a native female English speaker. The 40 animations represented one of 5 types of relative clause, 32 animations depicted 4 sentential complements, 32 animations represented four adverbial clauses and there were 10 catch items. Catch items are designed to detect those that showed a *yes* bias. A description of each of the animations is available in Supplementary File 1.

The relative clauses were all full bi-clausal relatives, each attached to the direct object of a transitive clause. The five types included subject (transitive and intransitive), object, indirect object and oblique. Object relatives were discourse relevant in that they had an inanimate head noun and a pronominal subject (see Kidd *et al.*, 2007). Pronominal subjects were also included in the indirect object and oblique clause structures, again to reflect structures used in natural discourse.

Sentential complements included four complement-taking verbs, three of which were mental state verbs (think, know, pretend) and one of desire (wish). Adverbial clauses included two temporal (before, after), one causal (because) and one conditional (if).

The test sentences were chosen on the basis of pilot work carried out by the first author, work completed by Diessel & Tomasello (2000); Diessel & Tomasello (2005) and research by Frizelle & Fletcher (2014) and Frizelle *et al.* (2017). Based on the British National Corpus, the sentences include high-frequency nouns and verbs. Vocabulary was also cross-referenced with the English MacArthur Bates Communicative Development Inventory (CDI; Fenson *et al.*, 2007) to ensure an early age of acquisition. Example test sentences for each structure are shown in Table 3.

The animations were shown in one of two standard random orders (forward/backward) to control for order effects. In previous work, we have used a 10 items for each structure, with a smaller range of structures. For the current study, we used eight animations for each structure i.e. each relative clause type, complement taking verb and adverbial clause, to avoid tiring children while testing a range of structures. Of the eight animations, four matched the structure and four did not. The design of those that do not match was dependant on the structure being assessed. In the case of relative clauses there was always an alternative to the head noun to which the relative clause was referring. For example, the representation of the sentence *He laughed at the girl he threw the ball to* included another girl in the animation who was holding a ball. Where the animations matched the given sentence, the action was carried out on the head noun as expected (in this case *the girl he threw the ball to*). However, when the animations did not match the sentence the action was carried out on the alternative (the other girl). Examples of correct and non-match items are available on YouTube, at https://youtu.be/d3dz_m8zTvc and https://youtu.be/FMxYzSyCs34 o respectively.

In the case of complement clauses non-match items were verb-dependant. Complement clause animations depict think/not think (the non-match item showing that the person in the animation has seen what has happened), know/not know (the non-match item showing that the person has not seen what has happened), pretend/not pretend (the non-match item showing that the person is using the object for what it is intended) and wish/not wish (the non-match item showing that the person already has the desired object). Examples of correct and non-match complement clause animations are available at https://youtu.be/OM27lMM4zPs and https://youtu.be/yPBQP14VjFA, respectively.

Regarding adverbial clauses, the non-match items for those that were temporal were shown in the order of the events depicted ( *before*/ *after*). For the adverbial *because,* non-match items were represented by depicting the event as it was described by both verbs, but not causally (e.g. for the sentence *The girl cried because the boy pushed her*, the animation showed a girl who was initially crying but then stopped before the boy pushed her). Finally for the conditional adverbial *if*, the non-match items were depicted as untrue/not if (e.g. for the sentence *If the gate was open the horse could walk away* the animation showed that the horse was tied up so that even if the gate was open he could not walk away). Examples of correct and non-match adverbial clauses are available at https://youtu.be/ILsCSUriGRU and https://youtu.be/Cd-EBpCtzZw, respectively.

Animations were on average 6 seconds in length. Children were simultaneously presented with each animation and a pre-recorded sentence orally. They were given the opportunity to hear each sentence-animation pairing more than once if needed, however this was rarely asked for. Children were asked if what was shown in the animation matched the sentence they heard and to respond by touching either the smiley or sad face on the Surface Pro 4 tablet touch screen. Total scores were calculated for each child and for each construction type. We used binomial theorem to establish that a total TECS-E score of 64 or above was significantly different from chance performance at a probability level of 0.01. When comparing success rates on different construction types, a score of 7 or 8 out of 8 items correct was scored as a ‘pass’ and a score lower than this as a ‘fail’. The probability of scoring 7 or more correct by guessing was computed by the binomial theorem as p < 0.036.

### Statistical analysis

All statistical analyses were performed using R Statistical Software ( R Core Team, 2018).

1) Internal consistency of TECS-E was calculated to give an index of reliability.
2) Binomial Theorem was used to establish a response threshold that was above chance for the TECS-E and TROG assessments.
3) Multiple regression analysis was used, in which total score on the sentence verification task was the dependent variable, and intellectual level (impaired/unimpaired) was the predictor variable. We then compared the two groups with intellectual disability and included age in the model with sentence verification as the dependent measure.
4) Hierarchical linear regression was used to determine the contribution of explained variance by predictors for the sentence-verification task (TECS-E) and TROG-2 (a multiple-choice comprehension task) respectively. In addition, we calculated the 95% confidence interval around the R ^2^ values in the regressions using the CI.Rsqlm function from the psychometric package in R. This was used to compare total explained variance of predictors in both dependent measures.

## Results

### Internal consistency of TECS-E

The alpha function of of the R psych package ( Revelle, 2018) was used to assess internal consistency of TECS-E for the whole sample, giving Cronbach’s alpha of .877.

### Children’s understanding of complex sentences – a comparison of the three groups

Our first hypothesis was that, based on the performance of young TD children, children with Down syndrome would be able to perform at above chance level on complex sentences when assessed using the sentence-verification task (TECS-E), designed to minimise extra linguistic and cognitive demands. Our results were contrary to our hypothesis, in that only 39% of the children with DS performed at a level above chance. This was in stark contrast to the two control groups, the majority of whom performed above chance level (74%) in the CI group and all of whom performed above chance in the TD group. Complete raw data for this study can be found on the Open Science Framework (OSF) ( Frizelle *et al*., 2018b)

**Figure 1.**
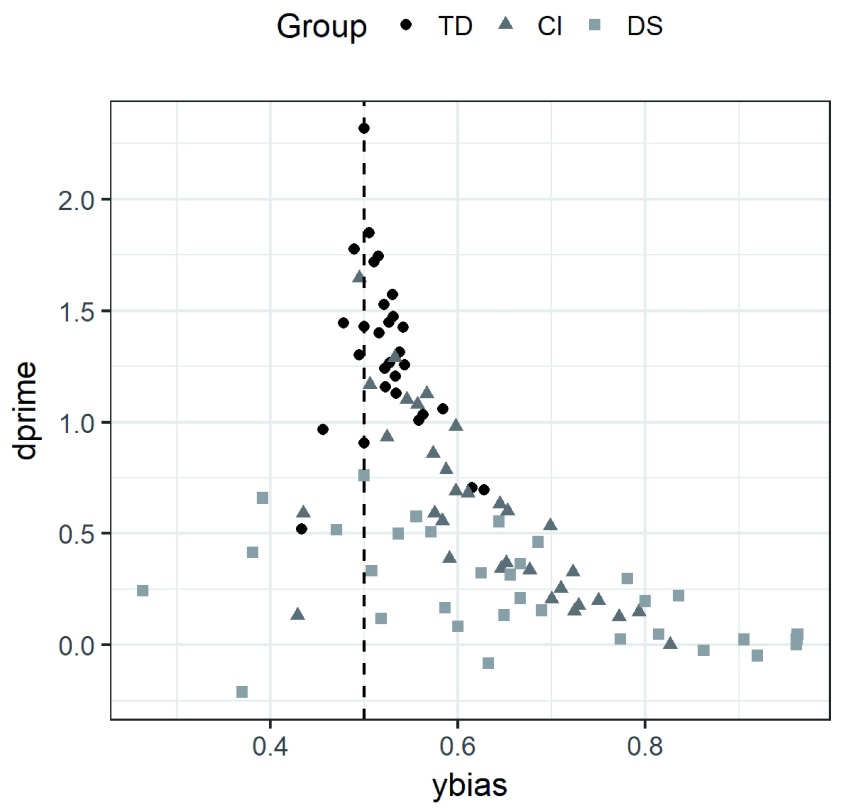
Plot of *d* prime measure against the proportion of correct yes responses (ybias).

There is the possibility of response bias when completing a task that requires a yes/no response, whereby the child may always give a ‘yes’ or ‘no’ response when they do not understand the construction presented. In order to examine this we converted the proportions of hits and false positives to a *d* prime measure ( McNicol, 1972) and plotted this against the proportion of correct ‘Yes’ responses (see Figure 1). If there is no response bias then the % correct that are ‘Yes’ should be around 50%. The plot shows clearly that for most children, when they do not understand, they are biased towards a ‘Yes’ response, however there are four children with DS who show a ‘no’ bias.

**Figure 2.**
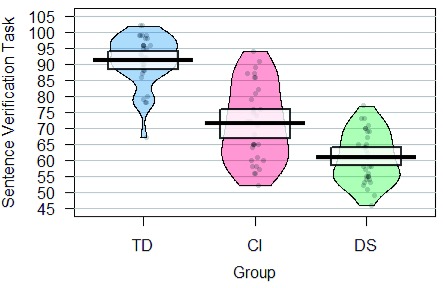
Pirate plot ( Phillips, 2016) of the performance of each group on the sentence verification task (TECS-E). The points represent the raw data (jittered horizontally), the bars show the means, with the surrounding rectangle showing the Bayesian 95% Highest Density Interval for the mean. Each dataset is enclosed by a smoothed density plot.

Our second prediction was that children with Down syndrome would perform at a similar level on TECS-E to those with cognitive impairment of unknown aetiology but at a lower level to those with typical development (matched on non-verbal mental age). We first performed a multiple regression analysis in which total score on the sentence verification task was the dependent variable, and intellectual level (impaired/unimpaired) was the predictor variable. In line with prediction, there were substantial effects of intellectual level, with the intellectually impaired children achieving an average score of 25.12 points less than the TD children ( *p* < 0.001), despite being matched on non-verbal mental age. In a second regression we compared the two groups with intellectual disability and included age in the model. Here, the results were contrary to our predictions in that there was a highly significant effect of DS. Children with DS achieved an average score of 10.25 less than the CI group ( *p* < 0.001) showing a disproportionate difficulty in their ability to understand complex sentences. The performance range of each group is illustrated in the plot at Figure 2.

Our third prediction was that children with Down syndrome would have greater difficulty understanding comparable constructions on the TROG-2 (a multiple-choice comprehension task), than on TECS-E. Here we could not make a statistical comparison as children with DS performed at floor on the TROG-2. We discuss this further in our descriptive analysis.

***Predictors of children’s performance on TECS-E.*** Our next prediction related to the factors that were associated with children’s performance on TECS-E. We hypothesised that the ability of children with DS to understand complex sentences would be explicable in terms of their deficits in verbal short-term memory, working memory and hearing level. To investigate this, we conducted a hierarchical linear regression analysis using likelihood ratio tests. The results are shown in Table 4. In the first step, mental age was entered in to the model in order to control for its effect on all children’s performance. This was followed by the memory variables (forward and backward digit accuracy scores) both of which were highly significant in accounting for a further 62% of the variance in TECS-E score. Note the accuracy scores were used for both forward and backward span tasks and although children were attempting to repeat the numbers in reverse order for the latter, the accuracy score does not take sequential order into account. For this reason, we are considering the backward span task to reflect verbal short-term memory rather than working memory. Finally, DS status was entered into the regression and although it only accounted for an additional 2% of the variance, the contribution was significant ( *p* = 0.011). This shows that children’s performance on TECS-E is not completely explained by their poor memory skills and that DS status makes an independent contribution to children’s performance on the task. There was insufficient variation in children’s hearing threshold; therefore, it was not added to the model.

**Table 4.** Hierarchical linear regression: predicting sentence verification score from mental age, memory ability and Down syndrome (DS) status.

Outcome variable (sentence verification score)	Predictor variables	Adjusted R ^2^	R ^2^ increase	Likelihood ratio test between subsequent models ( *p*-value)
Null	Mental age	0.051	-	
1	Mental age and forward and backward recall (accuracy score)	0.667	0.616	< 0.001
2	Mental age, forward and backward recall, DS (accuracy score)	0.686	0.019	0.011

The parameter estimates from the final model ( Table 4) can be found in Table 5.

**Table 5.** Parameter estimates from final linear regression: predicting sentence verification score from nonverbal mental age, memory ability and Down syndrome (DS) status.

Predictor	*b*	95% CI	*t(93)*	*p*
Intercept	53.29	*[38.13, 68.45]*	6.98	< .001***
Mental age	0.07	*[−0.10, 0.25]*	0.83	.411
Backward recall	0.70	*[0.35, 1.06]*	3.95	< .001***
Forward recall	0.59	*[0.26, 0.91]*	3.57	.001**
DS	-6.51	*[−11.50, −1.52]*	-2.59	.011*

Our final prediction relates to the question of whether cognitive and memory variables would account for more of the variance in children’s performance on the multiple-choice comprehension task (TROG-2) than on TECS-E. Given the additional executive demands of multiple-choice comprehension tasks ( Frizelle *et al.*, 2017) we anticipated that this would be the case. To test this prediction, we carried out a parallel regression analysis using the same independent variables as those previously described, but using TROG-2 raw score as the dependant variable. As before, mental age was entered into the model first to control for its effect. It accounted for 6% of the variance in TROG-2 score (r ^2 ^= 0.06 *p* = 0.009). This was followed by the memory variables, which accounted for a further 67% of the variance in total TROG score (both *p* < 0.001). Finally, the DS variable was entered into the model, accounting for a further 1.2% of the variance in children’s performance on this assessment. The results were in keeping with our prediction and are shown in Table 5. A key question was whether the prediction of TROG-2 scores by the mental age and memory measures was better than prediction of TECS-E scores: to check this, we calculated the 95% confidence interval around the R ^2^ values. The 95% confidence intervals overlapped (TECS-E: 0.576–0.775; TROG-2: 0.656–0.823), indicating that the difference in magnitude between the estimates was not reliable.

The parameter estimates from the final model ( Table 6) can be found in Table 7.

**Table 6.** Hierarchical linear regression: predicting TROG-2 scores from mental age, memory ability and Down syndrome (DS) status.

Outcome variable (TROG-2 score)	Predictor variables	Adjusted R ^2^	R ^2^ increase	Likelihood ratio test between subsequent models ( *p*-value)
Null	Mental age	0.060	-	
1	Mental age and forward and backward recall (accuracy score)	0.731	0.672	< 0.001***
2	Mental age, forward and backward recall, DS (accuracy score)	0.743	0.012	0.025*

**Table 7.** Parameter estimates from final linear regression: predicting TROG-2 scores from nonverbal mental age, memory ability and Down syndrome (DS) status.

Predictor	*b*	95% CI	*t(93)*	*p*
Intercept	9.96	*[−9.25, 29.17]*	1.03	.306
Mental age	0.09	*[−0.14, 0.31]*	0.77	.445
Backward recall	1.28	*[0.83, 1.73]*	5.68	< .001***
Forward recall	0.73	*[0.32, 1.15]*	3.52	.001**
DS	-7.27	*[−13.60, −0.95]*	-2.29	.025*

***Descriptive analysis of different clause types.*** Our final analysis as outlined in our pre-registered report was a qualitative/descriptive one. In this analysis we calculated the proportion of children in each group that passed each construction on the sentence verification task (shown in Table 8), where a pass was defined as a score of 7 or 8 out of 8 items correct. This allowed us to document the order of difficulty of the different complex sentence types in the three groups and to consider if the relative clauses followed the same rank ordering as was observed in our prior study of TD 3- to 5-year-olds ( Frizelle *et al.*, 2018a). An analysis of Table 6 shows that within each type of complex sentence (relative, adverbial, complement), all three groups performed best on relative clauses, while children’s performance on adverbial and complement clauses was similar within each group. With the exception of subject intransitive relatives, children with DS had significant difficulty understanding all other relative clause types. Their performance on adverbial and complement clauses was at floor (ranging from 0–12% of children passing these constructions). Children with CI of unknown aetiology also found subject intransitive relatives the least difficult construction to understand and their relative clause performance showed the following pattern: subject intrans > subject transitive = Indirect object > Object = Oblique. In relation to adverbial clauses they performed best on the causal adverbial *because* and found the conditional adverbial *if* the most difficult to understand. Complement clause performance showed the strongest understanding of *pretend* constructions, with *think* causing the greatest difficulty. With respect to children with typical development, we can see that they performed near ceiling on all relative clause types. In a previous study ( Frizelle *et al.*, 2018a), reporting on typically developing 3- to 5-year-olds, we reported the following hierarchy: intransitive subject > indirect object = transitive subject = object = oblique relatives (where ‘>’ refers to significantly greater than, and ‘=’ refers to no significant differences). However, the children included in the current study are considerably older, ranging in age from 5;01 to 7;09 years and we therefore expect a more stable performance across relative clause types. In relation to adverbial clauses, the current study shows that children with typical development had the greatest understanding of *before* temporal and *because* causal constructions, with the conditional *if* causing difficulty for 76% of these children. Within the complement clause constructions assessed, *wish* and *pretend* presented the least difficulty and *think* constructions were most difficult for children to understand.

**Table 8.** Proportion of children in each group that passed each construction.

Group	TD	CI	DS
**Relative clauses**			
Sub intransitive	0.94	0.66	0.39
Sub transitive	0.88	0.50	0.09
Object	0.88	0.41	0.18
Oblique	0.97	0.41	0.06
Indirect object	0.97	0.50	0.06
**Adverbial clauses**			
Before	0.85	0.22	0.09
After	0.61	0.31	0.00
Because	0.79	0.38	0.12
If	0.24	0.12	0.03
**Complement clauses**			
Think	0.36	0.03	0.03
Know	0.55	0.16	0.09
Pretend	0.79	0.44	0.03
Wish	0.82	0.22	0.00

TD, typically developing; CI, cognitive impairment of unknown aetiology; DS, Down syndrome.

Finally, in relation to participants’ performance on comparable relative clauses in the sentence verification animation task versus TROG-2 (a multiple-choice comprehension task) ( Bishop, 2003), we previously noted that a statistical comparison was not possible (see section titled *Children’s understanding of complex sentences – a comparison of the three groups)*, as children with DS performed at floor on the TROG-2. The comparable constructions included in both tests were the intransitive subject relatives (attached to a main clause object). These are deemed to be the earliest bi-clausal relative clause construction to emerge in young children’s expressive language ( Diessel, 2004) and the easiest for children to repeat ( Diessel & Tomasello, 2005; Frizelle & Fletcher, 2014) and to understand ( Frizelle *et al.*, 2018a). In order to compare the two testing methods fairly, we needed to adopt a stringent scoring of the TECS-E, where a pass is credited for perfect performance (8/8 two-choice items correct); the probability of achieving 8/8 by chance (p = 0.004) is the same as for getting all four four-choice items correct on TROG-2. No child from the DS or CI samples achieved this level of performance, and only five children (15%) from the TD group did this well. In contrast 26/33 (79%) of the TD children, 13/32 (40%) of the CI children and 6/33 (18%) of the DS children had perfect performance on the intransitive subject relatives on TECS-E. This is in line with our prediction that more children would pass these constructions on the sentence verification task than on TROG-2.

## Discussion

### The understanding of complex sentences in children with DS

Our current study aimed to investigate how well children with DS could understand complex sentences such as relative clauses, adverbial clauses and complement clauses. In contrast to previous studies in which standardized tests were used and both simple and complex constructions were reported on together, we designed a task that focused solely on complex sentences and that would allow us examine the relative ease or difficulty with which each construction type was understood. In addition, our task was designed to focus on children’s linguistic ability and to minimise the cognitive demands evident in assessments using a multiple-choice design, which are likely to disadvantage those with DS. Based on previous results from 3- to 5-year-old TD children, using this type of task, we hypothesised that children with DS would be able to perform above chance in their understanding of a range of complex sentences. However, contrary to prediction, we found that only a little over one third of children with DS performed above chance on these constructions, despite having a mean non-verbal IQ of 69. Our result is somewhat surprising given the complex syntactic expressive data reported on by Thordardottir *et al*. (2002) coupled with the fact that receptive language is usually superior to expressive, in this population ( Chapman *et al*., 2002; Laws & Bishop, 2003). Despite the fact that clausal complements and relative clauses were noted in the narrative samples of individuals with DS in the Thordardottir study, children in the current study had significant difficulty understanding all three types of complex sentences assessed. However, one key difference between the current study and that of Thordardottir and colleagues, which could account for this apparent superior expressive performance, is the age profile of the participants with DS in each study. Children in the current study ranged in chronological age from 6;10 to 11;08, with an average mental age of 6;7 years, while those in the Thordardottir sample were adolescents spanning a chronological age range of 12;5 to 20;4 years; we are not given information on their mental ages. In addition, we cannot assume a similar trajectory across receptive and expressive domains. A number of studies have shown that expressive grammar in people with DS continues to develop throughout adolescence and possibly into adulthood ( Chapman *et al.*, 2002; Laws & Gunn, 2004), whereas those exploring syntactic comprehension report mixed findings; many suggesting that syntactic comprehension is likely to reach a plateau in late adolescence or even to decline with age ( Chapman *et al.*, 2002). Although not specific to complex syntax, a recent study by Witecy & Penke (2017) found that receptive syntactic growth in those with DS continues through childhood into adolescence. Therefore, while our findings suggest that children with DS have significant difficulty understanding complex sentences at this point in their development (with an average mental age of 6;07 years), they may have the potential to understand them as they progress into teenage and adolescent years, with the corresponding increase in their cognitive functioning. A longitudinal study would be required to confirm this.

***A comparison of those with DS and the two control groups (matched on non-verbal mental age).*** In our second hypothesis, we predicted that children with DS would perform more poorly overall than TD children matched on non-verbal mental age but at a similar level to those with cognitive impairment of unknown origin. Again, our results did not support our prediction, in that the children with DS performed at a dramatically lower level than both control groups. Their significantly lower performance than the CI group shows that children with DS have a disproportionate difficulty in their ability to understand complex sentences even when compared to other children (matched on non-verbal mental age) who are cognitively impaired. Our findings contrast with those of a number of previous studies comparing people with DS and those with intellectual or language impairment. Using the TROG-2 as the receptive language measure, studies such as those by Finestack *et al*. (2013), DS versus Fragile X; Joffe & Varlokosta (2009), DS versus William syndrome, and Laws & Bishop (2003), DS versus SLI, have reported a similar performance between each group pair. However, there are two important distinctions between the TROG-2 ( Bishop, 2003) and the sentence verification task used in the current study. Firstly, the TROG-2 was not designed to focus solely on complex syntax and includes a range of syntactic constructions. Of the 20 blocks, 10 focus on simple sentences (for example, of the form SV, SVO, SVC and SVA), 1 block on reversible passives, 3 blocks on co-ordination, and the remaining 6 blocks focus on complex sentences. As is the case with most standardized measures that are designed for clinical use and not solely as research tools, children must show an understanding of the syntactically simple constructions before they progress on to those that are more complex. Therefore, by applying the discontinue rule, if a given number of items are failed, children will not be tested on complex sentences. Given the populations under scrutiny tend to have significant receptive language difficulties; it is probable that they were not assessed on the more complex constructions. If this were the case, their similar performance would have been based primarily on their understanding of simple syntactic constructions and this would account for our contrasting findings.

The other considerable difference between the TROG-2 and the sentence verification task used in the current study, is the design of the TROG-2 (a multiple-choice sentence picture matching task). As we highlighted previously, linguistic tests using these multiple-choice tasks are high in executive cognitive demands ( Frizelle *et al.*, 2017) and likely to disadvantage those with cognitive impairment of any aetiology (including those with DS, Fragile X, Williams syndrome etc). It is plausible that the multiple-choice design lowered the performance of each of the cognitively impaired groups participating in these studies, which may have masked any potential differences in the ability of each group to understand a range of sentence structures. In contrast, the sentence verification task is designed to minimise additional cognitive demands and may therefore be more sensitive to linguistic differences between the groups.

In addition to the studies that have used the TROG-2 as their measure of receptive language, there have also been two studies using the TACL-R ( Carrow-Woolfolk, 1985) comparing people with DS with those with CI of a different aetiology. Both studies report similar findings to those in the current study, whereby those with DS performed more poorly than those with CI of unknown aetiology ( Chapman, 2006) and those with Fragile X syndrome ( Abbeduto *et al.*, 2003). On the surface, one subtest from the TACL-R ( Carrow-Woolfolk, 1985) on which the authors reported (Elaborated Sentences) is particularly relevant to the current study, as it includes complex sentences in its target structures (approximately 50% of the constructions are complex). However, as outlined in relation to the TROG-2, standardized tests tend to use a developmental design, where simple sentences are presented before those that are more complex and a ceiling rule is usually applied whereby the test is discontinued following a pre-specified number of incorrect responses (in this case three). It is therefore likely that the participants were not tested on many complex sentences. The TACL-R also uses a multiple-choice sentence picture matching design, the implications of which are discussed below (see Multiple-Choice v’s Sentence Verification Task). In addition, the contradictory evidence in relation to syntactic growth ( Witecy & Penke, 2017) versus decline ( Chapman *et al.*, 2002; Laws & Gunn, 2004) as people with DS reach adolescence further complicates the comparison of findings. Therefore, despite evidence of a disproportionate difficulty for people with DS in each of the two aforementioned studies, it is difficult to compare their results with those found in the current investigation.

One possibility raised by Stojanovik (2018) is that TECSE might disadvantage children with DS because the response format would lead to high error rates in children who were biased to say ‘yes’. In order to rule out this possibility, we would need a comparison task with the same yes/no format, but where we were confident that the children understood the items. For instance, the child might be shown a picture of a girl drinking and just be asked to say ‘yes’ or ‘no’ to the sentence ‘a horse is drinking’. However, we also note that a high ‘yes’ bias could be a consequence rather than a cause of poor performance. One view of a child with poor comprehension of syntax is that their situation is similar to a competent adult who has to cope in a foreign country where they only have a weak grasp of the language: they will recognise individual words and create what sense they can from them in the context, but fail to understand more complex meanings conveyed by the word sequence. Someone in that situation is are more likely to say ‘yes’ than ‘no’ if they see a picture depicting all the components of the sentence, because it provides a good enough match to their patchy construction of meaning.

Our findings in relation to the TD group were in keeping with our hypothesis, in that children with DS performed at a significantly lower level than the TD controls. This result is consistent with previous findings (see Finestack *et al.*, 2013; Joffe & Varlokosta, 2009; Laws & Bishop, 2003; Pennington *et al.*, 2003; Price *et al.*, 2007; Rosin *et al.*, 1988).

***Multiple-choice versus sentence verification task.*** On the TROG- 2 (a multiple-choice comprehension task), both the DS and the CI group performed very poorly, with no children passing a block of items testing complex sentence comprehension; furthermore, only a minority of the TD children showed evidence of understanding these items. This was in stark contrast to performance on TECS-E, where the proportions passing 8 out of 8 items were 79%, 40% and 18% for the TD, CI and DS groups, respectively.

We should be careful not to over-interpret this finding, given that different sentences were used in the two assessments. Nevertheless, the results are consistent with a more controlled comparison of multiple-choice vs sentence verification methods by Frizelle *et al.* (2018a) with younger TD children. Even when identical sentences are used, it is never possible to equate items across different testing methods, because the multiple-choice method requires a set of distractors, depicting a range of different scenarios. Nevertheless, Frizelle *et al.* (2018a) found a consistent benefit for the sentence-verification method for young children, and we suggested this reflected the fact that sentences are presented in a manner more reflective of how we process language in natural discourse, with fewer processing and memory demands than in a multiple-choice test. By hearing the target sentence and seeing the animation at the same time, they can process the sentence in real time as they would in natural conversation. The presence of three distractors in TROG-2 requires the child to store in memory the arguments associated with each verb in order to rule them out. As we expected, our results show the impact of increased attention and memory demands to be particularly pertinent for the children with a cognitive impairment, who are likely to be disadvantaged using this assessment approach, in that the methodology is confounding their linguistic knowledge with other factors. For a more detailed discussion see Frizelle *et al.* (2017); Frizelle *et al.* (2018a).

***Predictors of performance on the TECS-E sentence verification task.*** In relation to the factors that would predict performance in children’s ability to understand complex sentences, our results, for the most part, are in line with what we predicted. Lack of variation in children’s hearing threshold meant we could not include this as a predictor variable in the model. However, in keeping with our hypothesis, verbal short-term memory was strongly predictive of children’s performance on TECS-E. In addition, DS status made an independent contribution to children’s performance, showing that children’s understanding of complex sentences was not completely explained by poor memory skills. Chapman *et al.* (2002) and Michael *et al.* (2012) have previously reported on the relationship between syntactic comprehension ability and memory in people with DS. Given our specific focus on complex sentences, which require the processing and integration of information from two clauses ( Martin & McElree, 2009), it is not surprising that we find memory to play an important role. More surprising was the independent contribution of DS status over and above poor memory skills, which indicates that the language difficulties of children with DS go beyond those usually associated with limited memory and nonverbal ability.

***Comparison of performance predictors on the sentence verification and multiple- choice tasks.*** In our final hypothesis we predicted that cognitive and verbal memory abilities would account for more variance on the multiple-choice than on the sentence verification animated task. We based our prediction on the aforementioned additional executive demands evident in multiple choice comprehension tasks ( Frizelle *et al.*, 2017; Frizelle *et al.*, 2018a). However, although the estimate of proportion of variance accounted for differed between the two tests, the confidence intervals of the estimates overlapped, and the estimates were high for both TECS-E and TROG-2.

***Performance of children with DS on different clause types.*** Our qualitative analysis revealed that of the three complex clause types, children with DS performed best on relative clauses, while their understanding of all types of adverbial and complement clauses was at floor. However, a closer inspection of the data showed that their superior understanding of relative clauses was skewed by their ability to understand one specific relative clause type, intransitive subject relatives, and only one-third of the children with DS showed some understanding of these constructions. With the exception of intransitive subject relatives, the children with DS performed at floor on all other relative clause types. The finding that intransitive subject relatives were the least difficult to understand (when compared to relatives including a range of syntactic roles) is in keeping with previous research findings in relation to children with DLD ( Frizelle & Fletcher, 2014) and children with typical development ( Diessel & Tomasello, 2005; Frizelle *et al.*, 2018a). This finding is also consistent with expressive acquisition data, ( Diessel, 2004) showing that when children start to produce full bi-clausal relatives, the majority are of the intransitive subject form.

## Conclusion

In summary, our findings suggest that despite using a method of assessment designed to minimise non-linguistic demands, children with DS have a disproportionate difficulty understanding complex sentences compared to two control groups matched on mental age. In addition, DS status made an independent contribution to how children performed on both the sentence verification (TECS-E) and multiple choice sentence picture-matching tasks (TROG-2) over and above their cognitive and verbal memory ability. This shows that these children’s understanding of syntax is not completely explained by poor cognitive or verbal memory skills (as measured here) and that a specific deficit understanding syntactic structures (even in children functioning in the borderline range of cognitive ability) may distinguish those with DS from other neurodevelopmental disorders.

## Data availability

Complete raw data for the study “The understanding of complex syntax in children with Down syndrome” is available on OSF. DOI: https://doi.org/10.17605/OSF.IO/X6UNE ( Frizelle *et al*., 2018b). Data are available on OSF under the CC-BY Attribution 4.0 International license.

## Software availability

The software source code for each of the experimental tasks is available on OSF under the CC-BY Attribution 4.0 International license. The DOI for each task is listed below.

- Forward digit recall task, DOI: https://doi.org/10.17605/OSF.IO/N9WMQ ( Duta, 2018a)
- Backward digit recall task, DOI: https://doi.org/10.17605/OSF.IO/B5N79 ( Duta, 2018b)
- Visuo-spatial memory task, DOI: https://doi.org/10.17605/OSF.IO/RVDXU ( Duta, 2018c)
- TECS-E complex syntax comprehension task, DOI: https://doi.org/10.17605/OSF.IO/AN7S2 ( Duta & Frizelle, 2018d)

With the exception of two of the TECS-E practice items, which are fully available, the source code for the remainder of TECS-E is uploaded with dummy videos to allow it to run. TECS-E needs to be normed and standardized before the complete assessment tool can be made available. Requests for access to the set of videos used in this study should be addressed to the corresponding author, with an explanation of why access to the videos is sought. It is not permitted to re-use them in any profit making endeavor. There are also examples of the videos available on YouTube (with links integrated in the Methods) to facilitate replication of the study.

## Supplementary material

**Supplementary File 1. Description of the videos included in the sentence verification task.**
