# Supplementary material for: The understanding of complex syntax in children with Down syndrome
Source: Wellcome Open Res. 2019 Feb 28;3:140. Originally published 2018 Nov 1. [Version 2] doi: 10.12688/wellcomeopenres.14861.2 (PMC6259485; doi:10.12688/wellcomeopenres.14861.2)
Supplement: Supplementary file 1 [file wellcomeopenres-3-16506-s0000.tgz › 396f10d3-8e6f-4ff8-a20f-5204f0eb89a1_Sentence_description_for_Open_Science.docx]

Sentence Verification task - Distractor description. *Denotes ‘yes’ response

**Relative Clauses**

| **Target Sentence** | **Animation** |
| --- | --- |
| **Subject Intransitive** |  |
| 1*. She followed the boy that ran away. | Two boys, one that ran away and one that stood still. A woman, who follows the boy, walking quickly after him. |
| 2*. She hugged the baby that cried. | Two babies sitting on the floor, one that cries and the other that does not. A woman, who picks up and hugs the baby that cried |
| 3*. She caught the boy that fell. | Two boys, one that falls from a tree and one that is standing at the bottom of the tree reading. A girl who catches the boy that fell. The girl also falls from the tree |
| 4*. He found the girl that was hiding | Two girls, one who is hiding and one who is playing with her toys? A boy that finds the girl that is hiding. |
| 5. She pointed to the man that was reading. | Two men one is sitting on a bench reading and the other is sitting on a different bench looking at a bird. A girl who is also sitting on a bench reading. She points to the man that is looking at the bird. |
| 6. She pushed the boy that was jumping. | Two boys, one jumping on a trampoline and one is standing holding a ball. A girl who is bouncing her ball. The girl pushes the boy who is holding the ball. |
| 7. He kissed the girl that fell. | Two girls, one that is walking and falls and the other is standing holding a cup. The boy kisses the girl that is standing with the cup. |
| 8. He pulled the dog that was eating. | Two dogs, one who is eating sausages and the other who is on a lead. A man is holding the dog on the lead, the dog pulls the man and the man pulls him back. |
| **Subject transitive** |  |
| 1*. He pointed to the girl that climbed the tree. | Two girls, one standing leaning against the tree and one that climbs the tree. A boy, who points to the girl that climbed the tree. |
| 2*. He saw the girl that picked the flowers. | Two girls one picking flowers and the other hiding behind the flower bush. The girl hiding looks up to see the other girl picking the flowers. A boy, who sees the girl picking the flowers but doesn’t see the other girl. |
| 3*. She pushed the man that was reading the book. | Two men standing on a bus, one is reading a book and the other is just standing. A woman, who pushes the man that is reading. |
| 4*. He chased the girl that took the ball. | Two girls and a boy holding a ball. One girl who takes the ball and runs away and the other who watches her. The boy chases the girl that took his ball. |
| 5. He followed the man that was riding the bike | Three men, two standing and one on a bike. The cyclist cycles in one direction. One of the men walks in the opposite direction and the third man follows him. |
| 6. He sat on the girl that was drinking the juice. | Two girls one sitting on the couch and one sitting on the floor. The girl on the floor is drinking juice. A boy (who is also drinking juice) sits on the girl on the couch. |
| 7. She wet the dog that scared the boy. | Two dogs, a girl and a boy. One dog that scares the boy. Another dog that interacts with the girl. The girl wets the dog that she is interacting with. |
| 8. She pulled the boy that was crossing the road | Two boys and a girl. One boy that crosses the road and one boy standing on the path, positioned to cross the road. The girl pulls the boy that is standing on the path. |
| **Object** |  |
| 1*. The girl found the shoe he lost. | A girl and a boy. Each standing on either side of a hedge. The boy is wearing one shoe. The other shoe is lost on the other side of the hedge. The girl finds the shoe. |
| 2*. The dog ate the banana he dropped. | A girl and a dog. The girl is eating a banana and drops it. The dog is standing beside her and there is another banana on the ground. When the girl drops her banana the dog eats it. |
| 3*. The man dried the plate she washed. | A man and a woman. There are dirty plates on the counter top. The woman washes a plate and gives it to the man. He dries the plate that she gave him. |
| 4*. The woman took the box he carried. | A woman with a box on the floor beside her. A man walks in carrying a box. The woman takes the box from him. |
| 5. The man dropped the cake she cut. | A man and a woman. The woman cuts a piece of a cake that is in front of her on a table. The man is holding a cake and drops it. |
| 6. The boy picked up the cup she broke. | A boy and a woman, each holding a cup. Both the woman and the boy drop their cups and they break. The boy picks up the cup that he broke |
| 7. The girl ate the sausages he cooked. | A girl and a boy. The boy is cooking sausages. The girl has some sausages in a picnic basket. She takes out her sausages and eats them |
| 8. The woman closed the bag that he opened. | A man and a woman. Each with a bag side by side on a counter top. The man opens the zip on one bag; the woman closes the zip on the other. |
| **Oblique** |  |
| 1*. The girl cleaned the teddy he played with | A girl and a boy sitting on the floor. The boy is playing with one teddy and there is another teddy on the floor beside him. The girl takes the teddy that the boy is playing with and cleans it. |
| 2*. The man built the wall she sat on. | A man, a girl and two walls. The man is building one wall. The girl comes and sits on the wall he is building. |
| 3*. The girl painted the wall he pointed to. | A girl and a boy. The girl is standing in a room painting one wall. The boy comes in and points at the same wall. |
| 4*. The boy dried the girl she through water at. | A boy and a girl sitting on a wall. Another girl carrying a bucket of water that she throws at the girl sitting down. The boy dries the girl who was sitting down, with a towel. |
| 5. The man opened the gate she jumped over. | A girl, man and two gates. The girl jumps over one gate. The man opens the other gate. |
| 6. The girl picked up the ladder he fell from. | A boy and a girl standing beside a tree. The boy climbs up a ladder leaning against the tree. He falls from the ladder. The girl is standing looking at him and there is a ladder beside her on the ground. The girl picks up the ladder that is beside her. |
| 7. The woman cleaned the wall he wrote on. | A man and a woman sitting in a waiting room. The man writes on one wall the woman cleans writing off a different wall. |
| 8. The girl wet the boy she took the box from. | A boy and a girl standing beside each other. The boy is holding a box. Another boy walks into the garden and gives a box to the girl. She takes it and then squirts water at the other boy. |
| **Indirect Object** |  |
| 1*. He kissed the woman he bought the shoes for | Two women and a man. One woman is trying on shoes the other woman is standing near the checkout. The man buys shoes, gives them to the woman standing near the checkout and gives her a kiss. |
| 2*. He followed the girl he gave the present to | Two girls and a boy. One girl is holding a present in her hands. The boy gives a present to the other girl. She walks away and he follows her. |
| 3*. She washed the baby she gave the bottle to | Two babies and a woman. The babies are sitting in the bathroom. The woman gives a bottle to one of the babies then lifts him up and puts him in the bath. |
| 4*. He laughed at the girl he threw the ball to. | Two girls and a boy. The girls are standing on either side of the boy. One girl is holding a ball. The boy throws a ball to the other girl, which hits her and he points and laughs at her. |
| 5. She kissed the boy she poured the juice for. | Two boys and a girl. The two boys are each holding a cup. The girl pours juice into one boy’s cup and then goes and kisses the other boy. |
| 6. She sat beside the man she gave the cake to. | Two men and a woman. The men are sitting on the couch watching television. The woman comes into the room with a slice of cake and gives it to one of the men. She then sits down beside the other man. |
| 7. She dried the boy she gave the ball to | Two boys and a woman. Both boys are wet. The woman gives a ball to one boy and then dries the other boy with a towel. |
| 8. She hugged the boy she showed the sandcastle to. | Two boys and a girl. The girl removes a towel from over a sandcastle and shows it to one of the boys. She then goes and hugs the other boy. |

**Complement Clauses**

| **Target Sentence** | **Animation** |
| --- | --- |
| **Think** |  |
| 1*. She thinks the cat is in the basket | A girl and a boy are in the garden with a basket in front of them in which there is a cat. The boy covers the basket by putting the lid on it and the girl leaves the scene. The cat pushes the lid off from inside the basket and jumps over the wall of the garden where it can no longer be seen. The boy puts the lid back on the basket. The woman comes back in to the garden again. |
| 2*. She thinks the boy’s hair is dry | A boy with wet hair is sitting on a toilet in a bathroom scene. A woman is drying his hair with a towel. When finished the woman walks past him to hang up the towel and as she does the boy throws a cup of water over his head to wet his hair again. The woman doesn’t see him. |
| 3*. He thinks the girl ate the cake | A boy and a girl are sitting on two chairs. The boy is holding a plate on which there is a slice of chocolate cake. He gives the cake to the girl and looks away. The girl takes the piece of cake and puts it behind the plant that is beside her. The boy looks back smiles and points at the empty plate. |
| 4*. She thinks the boy is up the tree. | A girl is standing in the garden watching a boy climb up the tree. She then covers her eyes as he climbs higher. The boy falls from the ladder, gets up, puts the ladder back against the tree and walks away. The girl uncovers her eyes and looks up in the tree to see where the boy is. |
| 5. She thinks the dog is in the box. | A boy and a girl are standing beside each other talking. The boy is holding a box with a dog inside it. The dog jumps out of the box and runs the way. The girl sees what happened. |
| 6. She thinks the box is empty. | As a girl walks onto the screen she sees a boy putting toys in a box. As she approaches the box she closes the lid and sits on it. |
| 7. She thinks the man ate her banana. | A older man and a girl are standing each with a banana in their hand. There is also a dog in the scene located between them both. As the girl goes to eat her banana it slips out of the skin and falls on the ground. The dog immediately eats the banana and she sees him eating it. She looks over at the man who then takes a bite from his banana. |
| 8. She thinks the girl took her toy. | A boy and two girls are standing outside. The boy is located between the two girls. One girl is holding a toy and the boy takes it from her. She hits him in anger and then walks away from the boy brushing past the other girl. |
| **Know** |  |
| 1*. He knows the girl broke the chair. | A girl sits on the chair and breaks it. A man is standing in front of her and saw what happened. |
| 2*. He knows the girl fell on the ground. | A girl is walking along the road, trips and falls. A boy is sitting on a bench with his skateboard and sees what happened. |
| 3*. She knows the boy ate the sweets. | A girl and a boy are sitting at a table with a bowl of sweets in front of them. The boy takes the sweets and eats them. The girl looks surprised having seen what happened. |
| 4*. She knows the boy broke the window | A boy kicks his ball towards a window and breaks it. The woman on the other side of the window sees what happened and shakes her fist at the boy. |
| 5. He knows the girl threw the ball. | A girl is on one side of a very high wall. A boy is sitting on the ground on the other side of the wall reading his book. The girl throws the ball over the wall. It lands beside the boy who looks surprised. |
| 6. She knows the boy is behind the door. | A boy is standing (hiding) behind a bedroom door. A girl comes into the bedroom and looks for him, under the table and under the bed. |
| 7. She knows the man took her dog. | A woman walks on screen with a dog on a lead. She ties the dog to a pole and walks away. A man walks on screen, unties the dog and walks away with him. The woman comes back on screen and looks surprised when she sees that the dog is gone. |
| 8. She knows the dog scared the baby | A dog jumps out from behind a television cabinet and frightens a baby who is sitting on a rug in the middle of the floor. The baby starts to cry and the dog runs away. A woman comes on screen and lifts the baby to comfort her. |
| **Pretend** |  |
| 1*.The girl is pretending she hurt her foot | A girl is standing in the grass. A boy walks on screen and as soon as he does the girl jumps up and down holding on to her foot. |
| 2*.The boy is pretending he is a pirate. | A boy takes a pirate hat, eye patch and sword out of a box. He puts them on plays with the sword as if getting ready to fight. |
| 3*.The girl is pretending the banana is a phone. | A girl takes a banana out of a fruit bowl that is located beside her on a table. She holds the banana up to her mouth (as though it was a phone) and pretends to talk into it. |
| 4*.The boy is pretending he ate the chocolate. | A boy and a girl are sitting at a table and there is a bar of chocolate on the table in front of them. The girl looks away for a moment at which point the boy takes the chocolate and puts it behind his back. The girl looks back and the boy starts to move his mouth as though eating. The girl looks surprised and then sad. |
| 5.The boy is pretending he is brushing his teeth. | A boy is standing in front of a sink. He picks up a toothbrush, puts toothpaste on it and brushes his teeth. |
| 6.The boy is pretending his slipper is a phone. | A boy is sitting on his bed with his slippers on the floor in front of him. He gets off the bed and puts on his slippers. He then picks up his phone and makes a call. |
| 7.The woman is pretending the box is a hat. | A woman is standing in front of a table with a box in front of her. She takes a hat out of the box and puts it on herself. |
| 8.The man is pretending he fell in the hole. | A man is walking along the road. There is a hole in front of him. He falls into the hole. |
| **Wish** |  |
| 1*. The girl wishes she had more presents. | A girl and a boy are each sitting in front of their respective Christmas trees. The girl is holding one present in her hand. The boy has a large number of presents. The girl looks over at all the boys presents, she then looks down at her own single present and starts to cry. |
| 2*. The boy wishes he was taller. | A boy is standing on a box and is trying to reach a container of biscuits. He can’t reach them. |
| 3*. The boy wishes he had more juice | A boy is standing by the side of a swimming pool. He has a drink of juice in his hand. He finishes the drink and then holds the cup up towards his mouth and empties out the last drop. He looks very disappointed when it is all gone |
| 4*. The woman wishes she had a trolley | A woman is in a shop and is having great difficulty trying to carry a basket full of groceries. An older man walks past with a large shopping trolley. |
| 5. The boy wishes he was on the swing | A boy is swinging back and forth on a swing. |
| 6. The girl wishes she had a dog. | A girl is standing in front of a house with a dog beside her. She rubs the dog’s head and puts it on a lead. |
| 7. The boy wishes he could climb the tree. | A boy is standing beside a tree and then climbs to the top. |
| 8. The girl wishes she could dress as a princess | A girl dresses up in a long dress, gloves, jewellery and a crown. |

**Adverbial Clauses**

| **Target Sentence** | **Animation** |
| --- | --- |
| **Before** |  |
| 1*.The boy played football before he watched TV. | A boy kicks and bounces his football. He then walks into a room and watches the television. |
| 2*.The boy jumped on the bed before the girl found her teddy. | A boy is jumping on a bed. A girl walks into the bedroom and finds her teddy on the ground under a pillow. |
| 3*. Before the boy kissed the girl he pushed her. | A boy and a girl are standing beside each other. The boy pushes the girl. She shows a sad facial expression. The boy gives the girl a kiss. |
| 4*. Before the girl fell from the tree she drank the juice. | A girl is sitting on the branch of tree with a juice carton in her hand. She drinks the juice and then falls from the tree, landing on her feet. |
| 5.Before the man washed his hands he brushed his teeth. | An old man is standing in front of a sink. The man turns on the tap and washes his hands. He then picks up his toothbrush and brushes his teeth. |
| 6.The man went into his house before it started to rain. | A man is standing outside the front door of a house. It starts to rain. He then opens the door and walks into the house. |
| 7.The boy fell before the girl threw the snowball. | A boy and a girl are outside in the snow. The girl picks up a snowball and throws it at the boy. The boy falls over. |
| 8.Before she brushed the boys hair she gave him a drink. | A girl is standing with a hairbrush in her hand. A boy is sitting down in front of her. She brushes his hair and then picks up a drink and gives it to him. He takes a drink. |
| **After** |  |
| 1*.The boy ate the cookie after he drank the milk. | A boy is sitting at a table and has a drink of milk. He then eats a cookie. |
| 2*. After the boy fell on the grass he scored the goal. | A boy runs towards a ball but fall on the grass. He kicks the ball while sitting on the grass and scores a goal. |
| 3*. After the man emptied the bucket the woman picked up the spade. | A man and a woman are standing in the garden. A man has a bucket full of leaves in his hand. He empties the leaves on the grass. The woman picks up the spade. |
| 4*.The woman cleaned the baby’s mouth after she washed his hands | A woman and a baby are sitting outside beside an outside tap. The woman turns on the tap and washes the babies hands. She then gets a cloth and cleans the babies mouth |
| 5.The girl opened the box after she put on her slippers. | A girl is standing beside her bed and there is a box on the bed. She opens the box and then puts on her slippers. |
| 6.After the woman put on her glasses she opened the door | A woman opens the door of a house and then puts on her glasses. |
| 7.The woman read the boy a story after she put on his boots | A woman is sitting on a park bench with a little boy beside her. She is holding a book and reads to the boy. His boots are on her lap. She puts the book down and puts the boots on the boy. |
| 8.After the baby started to cry the woman put on her headphones. | A woman is leaning against the back of a couch. She has an iPod in her hand and puts on a pair of headphones. A baby is sitting on a rug in front of her. The baby starts to cry. |
| **Because** |  |
| 1*.The baby cried because she spilt her milk. | A baby is sitting on the couch drinking a bottle of milk. She drops the bottle on the floor and the milk spills. She starts to cry. |
| 2*.The boy laughed because the clown fell over. | A boy and a clown are standing and there are some small balls in front of them. The clown starts to dance, slips on one of the balls and falls over. The boy points at him and starts to laugh. |
| 3*.The girl fell because the boy took her chair. | A girl and a boy are standing and there is a chair behind the girl. The girl goes to sit on the chair but as she does, the boy pulls the chair away. |
| 4*. Because she wanted the present the girl climbed on to the table. | A girl climbs up on to a table. She reaches up high to take a present from the top of a bookshelf. |
| 5.The girl cried because the boy pushed her. | A girl is leaning against a pole and is crying.  A boy walks up to her and pushes her. The girl looks surprised and stops crying. |
| 6.Because the woman took his ladder, the man fell. | A man is climbing up a ladder to straighten a picture on a wall. A woman is standing behind him. The man slips on the ladder and falls. The woman picks up the ladder and walks away with it. |
| 7.The girl crawled under the table because she wanted to get her teddy. | A girl is standing beside a table with a teddy in her arms. She crawls under the table, holding the teddy throughout. |
| 8.The boy cut his leg because he climbed the tree. | A boy walks into a small plant beside a tree. He cuts his leg and is bleeding. He then climbs the tree. |
| **IF** |  |
| 1*.If the boy was taller he could reach the teddy. | A boy is standing on a chair. He tries to reach a teddy, located on top of a cabinet. He can’t reach the teddy because he is too short. |
| 2*.If the woman put the baby down she could pick up the washing. | A woman is standing in front of a washing line holding a baby. There is a large basket of washing in front of her. She tries to lift the basket with one hand, but can only move it marginally, as it is too heavy. |
| 3*.The girl could carry all the toys if she had a bigger box. | A girl is standing in a room with toys around her, all over the floor. She has a small box under her arm into which she starts to put the toys. Having put 3 toys in the box, it is full. The girl walks away carrying the box with the 3 toys in it. |
| 4*.If the man moved his legs, the woman could sit down. | A man is lying stretched out on a couch with his legs on the armrest. A woman approaches him and moves his legs off the armrest but the man puts them back again. She appears annoyed and puts her hands on her hips. |
| 5.If the gate was open the horse could walk away. | A horse is in a field with his harness tied to a pole. The gate is closed. The harness is tight enough such that if the gate was open the horse could not walk out of the field. |
| 6.If the woman opened the box the girl could see what’s inside | A woman walks on to the screen with a closed box in her hand. She places the box on a high table. A little girl is standing beside the table and tries to jump up to see what is in the box. The height of the table is such that if the box was open the girl could not see what was inside. |
| 7.If the man had the ladder he could wash the window. | A woman is standing with a very small ladder by the wall of a high building. There is a window near the top of the building. A man walks on screen with a bucket and items needed for window cleaning. He looks over at the woman’s ladder and up at the window. The ladder is far too small to allow him reach the window to wash it. |
| 8.The baby could get her bottle if she was in her cot. | A baby is sitting on a bedroom floor. Her bottle is located on a chest of drawers such that if she was in her cot she could not get the bottle. |
