# Supplementary material for: The understanding of complex syntax in children with Down syndrome
Source: Wellcome Open Res. 2019 Feb 28;3:140. Originally published 2018 Nov 1. [Version 2] doi: 10.12688/wellcomeopenres.14861.2 (PMC6259485; doi:10.12688/wellcomeopenres.14861.2)
Supplement: Peer review history

# Peer review: Reviewer response for version 1

**DOI:** 10.21956/wellcomeopenres.16196.r34191

**Published:** 2018-12-20

**Authors:** Vesna Stojanovik

**Affiliations:** 1 School of Psychology and Clinical Language Sciences, University of Reading, Reading, UK

Thank you for inviting me to review this paper. I thoroughly enjoyed reading it and engaging with it.

 This study is a welcome contribution to the body of research on language comprehension in Down syndrome. It is well known that individuals with Down syndrome have difficulties with language acquisition and language is more of a challenge for individuals with Down syndrome compared to other populations of similar levels of cognitive impairment. The current study addresses a gap in the literature by assessing language comprehension in a fairly large group of children with Down syndrome (n=33) using a novel task which focuses on complex (subordination) syntactic structures (relative, complement and adverbial clauses). Children with Down syndrome's performance is compared to that of children who have similar level of non-verbal ability and a cognitive impairment of unknown etiology and a typically developing group of younger children who have similar non-verbal abilities to the Down syndrome group. Unlike other studies, the main task in the current study is a forced choice in which the children respond with yes or no. The results reported show that children with DS perform at floor on the understanding of all types of complex syntactic structures under investigation apart from intransitive subject relative clauses. In addition, the children with Down syndrome perform lower than both control groups and the presence of Down syndrome seems to explain significant amount of variance. The real strength of the paper is the carefully designed task to assess a range of complex syntactic structures involving subordination, the relatively large Down syndrome group and the detailed analyses of the results.

**Abstract:**

 This is well structured, but some points of accuracy need to be addressed .

 1) the three groups were not matched, but rather did not significantly differ in terms of non-verbal mental age. Also it needs to be specified that it was non-verbal cognitive ability (rather than leaving it vague as 'cognitive ability')

 2) in the Conclusions section, you need to be specific when you refer to mental age. I think you mean **non-verbal** mental age. 

**Introduction:**

 -the literature cited is current and relevant and presented and interpreted accurately.

 -I am a bit puzzled as to why only subordination was investigated, given that complex structures can be defined as those involving subordination and coordination. Given that you focus only on complex sentences which involve subordination, I think this should be made specific in the aims of the study.

 -Table 1 does not provide examples of each clause type.

 -The paper by Joffe and Varlocosta was published in 2007 and not in 2009 (the online version appeared in 2009 but the paper version came first in 2007).

 -You are basing your first hypothesis on a study by Thordardottir et al., 2002, who had much older participants in their sample. The ages of the children in your sample are closer to those in Joffe and Varlocosta (2007), although I know that this study doesn't specifically look at complement and adverbial clauses.

 -I totally understand the point that standardised assessments like the TROG-2 are discontinued after a certain number of failed blocks, hence in many studies individuals with Down syndrome have never been assessed on those blocks which tap the understanding of subordination and we don't know how well they can comprehend complex syntactic structures, However, if a child is struggling to comprehend simple SVA or SVO structures, what are the chances that they would comprehend clauses involving subordination? Is there any evidence fro m typical or atypical populations to show that this would be possible, i.e children do not comprehend simple structures like SVO but can comprehend complex clauses including different types of subordination?

 -The research questions and hypotheses are clearly stated.

**Methods:**

 The groups are of a good size and the power analysis shows that they are large enough for the proposed analyses.

**Results:**

 -Given that the majority of children with Down syndrome in this study (61%) were at or below chance on the TECS-E, is this something that needs to be elaborated on in the Discussion? Specifically, there seems to be quite a large response bias towards YES responses, so the question is how suitable this task is for children with Down syndrome?

 -When you discuss predictors of children's performance on the TECS-E and the hierarchical linear regression, and when you say that mental age was entered as a first step, do you mean verbal or non-verbal mental age?

 -Further below, when you entered the memory variables, you seem to have only considered verbal STM and WM, but not visuo-spatial?

 -Similar clarification on mental age and memory would be helpful when you discuss your final prediction (whether cognitive and memory variables account for more variance on the TROG-2 than on the TECS-E.

**Discussion:**

 -The Discussion addresses the hypotheses.

 -Typo: 1st paragraph, line 4 - "would allow us examine" should be "would allow us to examine".

 -I am wondering whether the TESC-E task was cognitively less demanding given that the children with Down syndrome performed almost at floor. What you seem to have shown in your study is confirmation of what some studies which had relied on standardised assessment measures have already implied and that is that children with Down syndrome may struggle or not be able to comprehend complex syntactic structures. 

 -Something for future research: if you want to find out whether having a yes/no task is cognitively less demanding than a task involving a choice of 4 pictures for children with Down syndrome, you need to look at a range of different structures (including simple and complex) using the 2 different tasks. By only focusing on complex syntax where the children were almost equally impaired in both tasks does not say much about task difficulty.

 One final comment: the interpretation of the data and the final discussion do not offer a clear theoretical explanation. There is a comment at the end that there may be a syndrome specific deficit in understanding complex syntactic structures in Down syndrome. I wonder whether adding a more theoretical elaboration of the findings (for example their implication for domain specific versus domain general theories of language acquisition) may strengthen the paper.

I have read this submission. I believe that I have an appropriate level of expertise to confirm that it is of an acceptable scientific standard.

# Response

**Published:** 2019-02-15

**Authors:** Pauline Frizelle

**Affiliations:** University College Cork, Ireland

We thank the reviewers for their positive evaluation of the paper, and for the suggestions they have made to improve it. We document here how we have responded to each one (with the exception of stylistic/typographical changes, which we have generally incorporated).

 Reviewer 1

 1.1. *Abstract reworded to cover the points raised*

 1.2 Focus on subordination

*In the introductory paragraph on complex syntax, we discussed co-ordination as well as subordination, but noted that our focus is on subordination. We now add a sentence to explain this, and are more explicit in the Aims section.*

 1.3 Table 1 does not provide examples

*Apologies. The wrong table was referred to. The tables have now be renumbered.*

 1.4 Reliance on the paper by Thordardottir et al. (2002) for their predictions despite difference in ages.

*Yes, we were cognisant of the fact that the participants in the Thordardottir study were chronologically older, however they may be more cognitively impaired than the sample in our study.  We also deemed it an appropriate study to refer to in the context of the structures that we are investigating. Very little work has been done exploring complex sentences involving subordination in this population.*

 1.5 Joffe and Varlokosta year of publication

*We have corrected the year of publication in relation to the Joffe and Varlokosta paper*

 1.6 If children fail simple structures on TROG, they are unlikely to pass complex ones.

*We agree. The point we were trying to make was simply that the stopping criterion might make TROG insensitive for revealing subtle differences between groups because of limited range.*

*1.7 Bias to 'yes' responses.*

*We now say a bit more about this in the Discussion, noting (a) how further testing with additional very easy items could help distinguish whether a 'yes' bias was a general tendency of the child; and (b) considering why a 'yes' bias is likely to be the default response for a child who does not fully understand.*

 1.8 Use of term 'mental age'

*We have clarified that when discussing our own study we are referring to nonverbal mental age throughout the paper.*

 1.9 why is visuospatial memory not included?

*The inclusion of Visuo-spatial STM in the method was as a positive control (to show that our results would be regarded as typical of this population and that there wasn’t some anomaly with our particular group of participants). When we pre-registered the study we outlined our analyses in detail and did not power the study to include VSTM as a predictor variable.*

 1.10 Question regarding whether the TESC-E task was cognitively less demanding given that the children with Down syndrome performed almost at floor.

*Although most of the children with DS performed at floor we do see emerging understanding of the intransitive subject relatives. This is in keeping with young typically developing children who demonstrate an understanding of this construction before relative clauses that have other syntactic roles. Our previous work suggests that TECS-E is a less cognitively demanding task – we refer to the two studies below.*

 Frizelle, P., Thompson P.A., Duta, M. & Bishop, D. V. M. (in press) Assessing children’s understanding of complex syntax: a comparison of two methods. *Language Learning*. Pre print available at https://osf.io/27gwb/ DOI 10.17605/OSF.IO/27GWB

 Frizelle, P., O'Neill, C., & Bishop, D. V. M. (2017). Assessing understanding of relative clauses: a comparison of multiple-choice comprehension versus sentence repetition. *Journal of Child Language*, 1–23. http://doi.org/10.1017/S0305000916000635

 1.11 Suggestion re the need to include simple and complex sentences in future research to determine whether having a yes/no task is cognitively less demanding than a task involving a choice of 4 pictures for children with Down syndrome.

*We agree that it would be helpful to look at both simple and complex sentence when comparing assessment methods in the future. However, our work with typically developing children shows that the design of distractors currently used in standardized tests (such as TROG-2, ACE) using a multiple choice format (when assessing complex sentences) is particularly problematic and is disadvantageous to those with difficulties in executive functioning. the design of distractors required to assess simple sentences may not be as problematic.*

 1.12 Theoretical discussion

*We appreciate the suggestion to strengthen the paper by situating the results in the context of domain-specific vs domain-general theories of language development, but we have not taken this step because we are concerned that findings from atypical groups can be misleading. The problem is that something that looks like a rather pure and specific syntactic deficit can be the consequence of a downstream problem with a specific sensory or motor system very early in development. See Bishop, D. V. M. (1997). Cognitive neuropsychology and developmental disorders: uncomfortable bedfellows. Quarterly Journal of Experimental Psychology, 50A, 899-923. We feel that studies such as this one can help us disentangle the role of different cognitive systems on language development, but that accounts of language as domain-specific vs domain-general are too polarized.*

 ​​​​​​​

# Peer review: Reviewer response for version 1

**DOI:** 10.21956/wellcomeopenres.16196.r34208

**Published:** 2018-11-27

**Authors:** Nicola Botting

**Affiliations:** 1 Language and Communication Science, City, University of London, London, UK

Summary

 Overall this is an interesting and well written paper which addresses some in depth questions regarding the language of children with Down Syndrome (DS).  Previously studies have shown that people with DS have better receptive than expressive language and that vocabulary is better than syntax.  This study presents important information on the understanding of complex sentences, and what other factors might predict this skill. The study has many strengths including the introduction, comprehensive assessment battery and careful procedure, as well as the large number of participants compared to much of the literature of this kind. 

 My main comments are around a shift in the paper from working memory to short term memory which seems to be driven by the use of a non-sequential  backwards span score. Similarly, although a visual STM task was administered, it is not then used subsequently. Given the fact that verbal STM may be influenced by language skill (as well as the other way around), it would be interesting to know how non-verbal STM associated with syntactic scores and to discuss this.  This may also help to make sense of the finding that VSTM did not explain more language variance using TROG compared to the TECS-E.  It may be worth adjusting parts of the paper, including the overall interpretations, with these issues in mind. 

 I have detailed a number of comments below which I hope will be useful and enhance the paper further.

Abstract

 p.2 In fact hearing level was not really considered (for good reason) and should maybe be taken out here.

 p.2 The term impact is causally loaded.  It may be that sentence processing impacts on cognition and memory.

Introduction

 p.3 It would be useful to have some examples.  The text says that examples are shown in Table 1 but in fact this is a summary of studies.  May be there is a table missing here? Or is it Table 3 later?

 p.4 The critique of the TROG-2 format is also relevant to most other groups with poor language. i.e. many children with DLD also show inattention and limitations in array scanning, which raises interesting wider questions about assessment of this type.

 p.4 It might be interesting to raise the possibility of Dynamic Assessment here, which has been shown to be a reliable method of assessing early language.

 p.4 The sentence beginning ‘Although…’ might read better as ‘Although it is tempting to consider cognitive ability as a core….’.

 p.5 It would be useful to give a sentence or two summarising whether the authors think Table 1 does indeed show a unique profile of language for those with DS or not. At the moment it just says that it is possible to see whether there is a distinctive profile.

 p.5 The authors introduce working memory here, but later do not use the assessments as WM measures in analysis.  It might be useful to flag up the complexities of WM here?

 p.5 I expected to see Hick et al. (2005) here (visual STM =TD >DLD and Verbal STM=DLD p.5 Should read ‘(Frizelle et al 2018a)’ – no gap between 2018 and a.

 p.5 It would have been helpful to have some justification for the prediction that *preten*d would be easy and *think* difficult.

 p.6 Hypothesis 4 includes working memory even though backward span was included as a STM task.

Methods

 p.6 The power analysis section might be better at the start of methods?

 p.6 The sentence ‘We had anticipated…’ is difficult to read.

 p.7 Backward digit span is described as an executive task, but this is slightly misleading since it is not used this way in the analysis. A note here commenting that the accuracy score does not give a WM measure would be helpful.

 p.7 In general, some adjustments have been made to digit recall, which are all small but which may also add up: accuracy scores / additional practice trials /option to change one’s mind.  A comment on this later in the paper might be warranted.

 p.8 Some more information would be useful about the TECS-E in relation to test-retest reliability and internal consistency.

Results

 p.10 OSF needs explaining in full on first mention.

 p.10 I could not easily match the text with the figure – at what threshold are authors considering responses to represent a ‘yes’ or ‘no’ bias.  There are 5 DS children below 50% and 4 below 40%  but the text says ‘a couple of DS children’.  Apologies if I have misunderstood the graph but if so, I suspect others may also.

 p.10 Full statistical details for regressions 1 & 2 would be useful.

 p.10 It would be helpful to know how many children scored at floor on complex constructions.  Did all the children score 0?  If not could the data be analysed categorically as did/did not score 0?

 p.10 Working memory is again listed as a predictor, but is later followed by a statement on p.11 saying the measure is not WM.

 p.11 Fig2 would benefit from stating that this is the TECS-E on the axis and/or in the title.

 p.11 Here I expected the block recall task to appear in the hierarchical models. Given that VSTM may be mediated by language ability, it would be interesting to know how non-verbal memory associates with the TECS-E.  There may be a robust reason not to include it, for example maybe because this is a relative strength, but this information would be helpful to the reader since the measure is included in the methods.

 p.11 The term qualitative is misleading here as this analysis uses numbers and is not ‘qualitative analysis’ in the way the term is often used.  Perhaps ‘descriptive’ would be better suited?

 p.11 The proportion of children passing adverbial and complement clause items is given as 0-9%.  However, in Table 6, the adverbial 'because' shows a 12% pass rate.  This may be because authors are later considerate of 'because' being an exception, but that may need explaining here.

 p.12 Authors might consider reporting the constant value in hierarchical regression models for completeness.

 p.12 The sentence ‘In relation to…’ was ambiguous on first reading and sounded as if it meant ‘In comparison to…’.  Maybe change to ‘For children with…’ or ‘With respect to children with…’.

 p.12 It would be worth emphasising here that the Frizelle study involves typically developing children.  It also feels a little out of place to give this much detail from another study in a results section.

 p.12 Again ‘(2018 a)’ should be ‘(2018a)’.

 p.12 The sentence ‘For a fair comparison…’ is not very clear and could be reworded.

Discussion

 p.13 At this point I began to think about the WM content of the Leiter, and also of assessments used in other studies to define cognitive impairment.  The BPVS, TROG and other multiple choice format assessments might particularly disadvantage groups who have been defined as cognitively impaired using tests which tap into this weakness.  The authors might want to raise a point about classification of CI more generally in research.

 p.14 The argument that the TECS-E has fewer cognitive demands does not seem supported by the comparison of regression data, where variance explained by STM was similar whether the TROG or the TECS-E was used.  I realise that the DS group were more able to access the TECS-E but given the overlap between VSTM and complex language in general (regardless of assessment) maybe there are explanations for this result which could be discussed. Perhaps for example Visual WM (or domain general capacity) would have shown differential relationships for the two assessments? Perhaps the TECS-E feels more naturalistc and is therefore more sensitive? It would be interesting to have some thoughts on this within the discussion.

 p.14 Similarly, the statement that language difficulties of children with DS go beyond those associated with memory could be adjusted to say verbal short term memory since visual memory and WM are not included in the analyses and could well soak up the remaining variance represented by the group variable.  

 p.15 The conclusion also states ‘…understanding of syntax is not completely explained by poor cognitive or memory skills’.  Again this could usefully read VSTM skills and possibly also say ‘as measured here’.  A wider mention of the usefulness of digit span accuracy as a memory task might also be worth discussing earlier.

 Many thanks for the opportunity to read and review this paper which will make a contribution to the field and will be read by academics and clinicians alike.

I have read this submission. I believe that I have an appropriate level of expertise to confirm that it is of an acceptable scientific standard.

# Response

**Published:** 2019-02-15

**Authors:** Pauline Frizelle

**Affiliations:** University College Cork, Ireland

2.1 Hearing level removed from abstract

*Have reworded 'impact' to 'associated with'.*

 2.2 No examples in Table 1

*Apologies. This was a labelling error also noted by the other reviewers. We have corrected it.*

 2.3 The relevance of difficulties with the multiple choice format for other groups with poor language e.g. DLD

*We agree and have made this point in relation to multiple choice sentence picture matching tasks, in previous work (see Frizelle, Thompson, Duta & Bishop, 2018; Frizelle, O’Neill & Bishop, 2017).*

 2.4 Dynamic Assessment

*While we agree that dynamic assessment is of interest in this context, we decided against introducing it here, because it might give readers the misleading impression that our study used it.*

 2.5 Question regarding whether the authors think Table 1 does indeed show a unique profile of language for those with DS or not.

*We have rephrased and added the following line 'While previous reports appear mixed and are somewhat dependant on the comparison group under scrutiny, previous literature suggests that children with DS perform at a similar level to those with Williams syndrome and specific language impairment.'*

 2.6 Complexities of working memory

*We have added some clarification regarding what is involved in working memory tasks and given an example.*

 2.7 Justification for prediction that pretend would be easy, think more difficult

*We have added this justification.*

 2.8 The inclusion of working memory in Hypothesis 4 even though backward span was included as a STM task.

*The study was preregistered and with extra practice items we had hoped to use backward span as a working memory task. The children’s performance was such that it made more sense to use it as an STM task.*

 2.9 Positioning of the power analysis

*We have moved the power analysis to the beginning of the methods section as suggested.*

 2.10 Description of backward digit span as an executive task but not used in this way in the analysis

*We have added a note with respect to the implication of using an accuracy score for backward digit span*

 2.11 Comment on small adjustments made to digit recall task

*We appreciate that a few small changes can add up to something more significant, however, we were not calculating standard scores or comparing to published norms. The test was administered consistently across the three groups so that all children had the same experience. In addition the pace at which participants heard the numbers was consistent throughout, this is something not controlled for when span tasks are administered without the support of an electronic device. Children are also permitted to change their mind when the WMTB-C is administered in its standard form.*

 2.12 p.8 Some more information would be useful about the TECS-E in relation to test-retest reliability and internal consistency.

*We do not have test-retest data, but do now report internal consistency (Cronbach's alpha).*

 2.13 Matching text with figure in relation to yes/ no bias

*We have altered the text from ‘a couple of DS children’ to ‘4 DS children’ those scoring between the 40- 50 range are not considered to show a distinct bias.*

*2.14 Full statistical details for regressions 1 & 2 would be useful.*

*These have now been added*

 2.15 Did all the children scoring at floor score 0? If not could the data be analysed categorically as did/did not score 0?

*Because participants had a 50% chance of getting the answer correct for each item on TECS-E we needed to establish what was chance performance in relation to a total TECS-E score. Given the yes bias it was highly unlikely that anyone would actually score 0. We believe that analysing children’s performance according to chance levels was a more appropriate reflection of children’s ability to do the task.*

*‘We used binomial theorem to establish that a total TECS-E score of 64 or above was significantly different from chance performance at a probability level of 0.01. When comparing success rates on different construction types, a score of 7 or 8 out of 8 items correct was scored as a ‘pass’ and a score lower than this as a ‘fail’. The probability of scoring 7 or more correct by guessing was computed by the binomial theorem as p < .036’*

 2.16 *When referring to working memory as a predictor we have clarified our use of the term by adding in ‘accuracy score’ in parenthesis.*

*2.17 We have added TECSE to the title of figure 2*

 2.18 Question re why block recall task did not appear in the hierarchical regression models

*The inclusion of Visuo-spatial STM in the method was as a positive control (to show that our results would be regarded as typical of this population and that there wasn’t some anomaly with our particular group of participants). When we pre-registered the study we outlined our analyses in detail and did not power the study to include VSTM as a predictor variable.*

*2.19 *Use of the term qualitative analysis misleading

*We have changed this to ‘descriptive’.*

 2.20 The proportion of children passing adverbial and complement clause items in text (when referring to causal adverbials) is not in keeping with table

*This was an error. The 0- 9% has been corrected to 0- 12%*

 2.21 Authors might consider reporting the constant value in hierarchical regression models for completeness.

*Now added.*

 2.22 Note that Frizelle study was with TD children- too much detail from another study in the results section.

*We have reiterated that in the previous Frizelle study we refer to TD children. We believe the reviewer may have thought some of the detail given referred to this previous study and have added clarification re when we are referring to the current study.*

*2.23 *The argument that the TECS-E has fewer cognitive demands does not seem supported by the comparison of regression data, where variance explained by STM was similar whether the TROG or the TECS-E was used.  I realise that the DS group were more able to access the TECS-E but given the overlap between VSTM and complex language in general (regardless of assessment) maybe there are explanations for this result which could be discussed. Perhaps for example Visual WM (or domain general capacity) would have shown differential relationships for the two assessments? Perhaps the TECS-E feels more naturalistic and is therefore more sensitive? It would be interesting to have some thoughts on this within the discussion.

*In our pre-registration visual WM was not included as part of our research questions/ hypotheses and therefore we are reluctant to include it post hoc. We had discussed task demands with respect to TECS-E and 1) multiple-choice sentence picture matching tasks (not specifically TROG) and 2) sentence recall tasks in two previous papers and did not want to be overly repetitive here. We now refer the reader to these papers for a more detailed discussion.*

*2.2.4 *Adjustment of statement that language difficulties of children with DS go beyond those associated with memory could be adjusted to say verbal short term memory (since visual memory and WM could well soak up the remaining variance)

*We have added the word ‘verbal’ in relation to the memory skills as we do not include visuo-spatial memory in the regression models. We have stated that because we have used ‘accuracy score’ in relation to the backward digit span task, we are not considering this to be a measure of working memory. However, we do believe that the very process of trying to recall a number of digits in reverse order does alter this task from one of straightforward span.*

**

*2.2.5 We have qualified our statement ‘…understanding of syntax is not completely explained by poor cognitive or memory skills’ by adding the word ‘verbal’ and the phrase ‘as measured here’.*

# Peer review: Reviewer response for version 1

**DOI:** 10.21956/wellcomeopenres.16196.r34171

**Published:** 2018-11-20

**Authors:** Christopher Jarrold, Nikolitsa Stathopoulou

**Affiliations:** 1 School of Experimental Psychology, University of Bristol, Bristol, UK; 2 School of Psychological Science, University of Bristol, Bristol, UK

Language acquisition is an area of special challenge for individuals with Down Syndrome. Many studies of both language comprehension and production have been conducted in an attempt to account for the language difficulties observed in children with the condition. However, as the authors note, the question of complex syntax understanding in Down syndrome is one that deserves further investigation; researchers believe that receptive and expressive syntax is an area of relative weakness but are still not clear on the extent of difficulties in this area. The purpose of the current article is to investigate the comprehension of complex syntactic constructions, including relative clauses, complement clauses (that serve as the object of the main clause), and adverbial clauses in children with Down syndrome by considering how memory and hearing ability influence the processing of these grammatical structures. Specifically, by using a newly devised animation task, Frizelle and colleagues test three groups of children, namely, one group of 33 children with DS and two control groups composed of i) 32 children with cognitive impairment of unknown aetiology (CI) and ii) 33 children showing typical development. These comparison groups were broadly equated to those with Down syndrome on a non-verbal measure of mental age. Overall, the results showed that the DS group performed more poorly on the majority of the test sentences than both control groups. The authors suggest that their findings cannot be explained by poor memory or other cognitive skills, but rather indicate a more specific syntactic deficit that distinguishes children with Down syndrome from other individuals.

 Overall the paper is very well-written. The sample sizes for this work are good, the use of an animated syntax comprehension task has potential benefits, the measurement of hearing ability is both novel and welcome, and the preregistration of study hypotheses and availability of data are obvious further strengths. The literature review is also well informed, but the authors may be a touch too reliant on the paper by Thordardottir et al. (2002) for their predictions. That previous study used narrative data, which may not be representative of conversational performance, and Thordardottir and colleagues themselves noted that MLU may not be a suitable measure of language complexity for older individuals. There is also work from non-English speaking populations with Down syndrome that the authors could include ^1^^-^^3^.

 It is somewhat unfortunate that individuals with Down syndrome perform so poorly on the TECS-E task that we learn relatively little about whether their profile of strengths and weaknesses across different types of complex syntactic structures is typical or atypical. The data indicate that, at the very least, comprehension of complex syntax is markedly delayed in Down syndrome, seen here relative to two groups of broadly comparable non-verbal abilities. The authors put forward good arguments for why the TECS-E task represents an advantage over tasks such as the TROG, and their data support these claims in showing more evidence of ‘success’ on aspects of their new measure than on corresponding blocks of the TROG.

 However, at the same time we wonder whether there may be some advantages to the TROG format that aren’t present in the TECS-E task. In particular, might it be possible that providing concrete representations of response options at test makes aspects of a grammatic comprehension task easier, perhaps particularly for individuals with Down syndrome? In a sense the TROG is more like a recognition task, and the TECS-E more like a recall task. Given this, one can potentially ask whether providing response options at test might allow individuals who have generated something like an incomplete representation of the utterance to make a sensible best guess at the answer and show some degree of competence. Again, we note that the current data provide no evidence for this suggestion, but our own anecdotal experience of working with participants with Down syndrome is that individuals often seem to struggle with the concept of ‘same vs. different’ and sometimes appear to have particular problems on tasks that require them to make such judgements. A related point is that if aspects of the task are difficult for individuals with Down syndrome, for whatever reason, then it is possible that they might give up on the whole task to some extent. The procedure isn’t completely clear on this point, but we assume that different sentence structures were interleaved with each other throughout the task. Work by Wishart ^4^ suggests that motivational issues might particularly affect task performance among individuals with Down syndrome, and the yes bias which the authors extract so elegantly from the data might potentially reflect a response to overall task difficulty among individuals with Down syndrome which could affect performance on even the easier constructions.

 In other words, before concluding definitively that individuals with Down syndrome have specific difficulties with the comprehension of complex syntax it would be good to check that there are not aspects of the TECS-E procedure which are not particularly difficult for this population to understand or respond to, and which might contribute to their apparent problems on the task. Future work with this form of procedure might usefully include simple structures where one would expect individuals to perform well, or ‘acting out’ tasks that require the participant to recreate the meaning of the utterance with toy figures. Alternatively, there may be aspects of the current data that can be used to show that individuals with Down syndrome do understand the basic logic of the task and were engaged with it – we note that a reasonable proportion of group ‘pass’ the sub intransitive relative clause items, and it might also be that the 10 catch trials could provide data relevant to this issue. Might it also be possible to examine the profile of performance of the subset of individuals with Down syndrome who appeared to clearly understand the TECS-E task?

 Our other substantive points on the paper relate to other aspects of the authors’ hypotheses, but we make these points in the knowledge that the authors’ commendable decision to pre-register aspects of their study may understandably have limited their willingness to conduct post-hoc analyses:

 The data clearly show that individuals with Down syndrome perform less well on the TECS-E than participants in the CI group. However, it may not be quite correct to suggest, as the authors currently do, that the three participant groups are ‘matched’ for non-verbal mental age. The p value for this comparison is not significant ( *p* = .131), and as many in the literature have noted, finding a non-significant difference on a ‘matching’ measure is not the same as showing that groups are equated on that measure. Table 2 shows a slight tendency for non-verbal mental age to be higher in the CI group, and while it seems highly unlikely that this tendency towards a difference drives all of the group effect on TECS-E performance, it might be worth including non-verbal MA as a covariate in the test of prediction 2.

 Similarly, while hypothesis 4 asks questions of the predictors of performance in the Down syndrome population, the corresponding analysis begins by analysing performance across the whole sample (see Table 4). Including group membership as a final step in this regression is not inappropriate, but because verbal short-term memory performance is clearly associated with group (see Table 2), entering this verbal short-term memory on the preceding step will have two effects.  First it risks reducing the effect of group on the final step (though this is already significant). Second, it may suggest an association between memory and performance which is driven by extreme group differences and which is not reflective of the predictors of task performance in any group in isolation (a version of the ecological fallacy noted by Robinson, 1950 ^5^). It may not be possible to carry out separate regression analyses for each group because of concerns about sample sizes and floor effects among the Down syndrome group, but it would be interesting to know whether verbal short-term memory and mental age are associated with performance on the TECS-E in just typically developing children.

 A more minor point is that it while it might be correct to call the Leiter-3 a test of cognitive ability (p. 8), because this is only a single test it might be over stating things to imply later in the paper that the analyses capture the contribution of cognitive ability to syntactic understanding.

 Finally we note some very minor typographic issues:

 On page 3 the authors state that ‘example of each clause type are given in Table 1’. In fact it is Table 3 that provides these example.

 On page 9 it is suggested that the probability of scoring 7 or 8 out of 8 trials of the TECS-E is less than .035. The exact probability is .0352, so less than .036 would be more accurate.

 Page 14 – should Chapman & colleagues (2002) instead be Chapman et al. (2002)?

 The references for Fortunato-Tavares et al. (2015) and Fowler (1990) papers are not in the correct position, alphabetically, in the reference list.

We have read this submission. We believe that we have an appropriate level of expertise to confirm that it is of an acceptable scientific standard.

## References

[1] ChristodoulouCGrohmannK: From First Steps to Full Acquisition: Comprehension of Subjunctive Clauses in Bilectal Children With Down Syndrome and Typical Language Development. *Frontiers in Communication*.2018;3: 10.3389/fcomm.2018.00019 10.3389/fcomm.2018.00019

[2] StathopoulouN: The Linguistic Profile of Greek Individuals with Down Syndrome: Evidence from Syntactic and Morphological Phenomena. *Ph.D. thesis, University of Essex, UK*.2009;

[3] StathopoulouNClahsenH: The perfective past tense in Greek adolescents with Down syndrome. *Clin Linguist Phon*.2010;24(11) : 10.3109/02699206.2010.511405 870-82 10.3109/02699206.2010.511405 20964505

[4] WishartJGDuffyL: Instability of performance on cognitive tests in infants and young children with Down's syndrome. *Br J Educ Psychol*.1990;60 ( Pt 1):10-22 214051610.1111/j.2044-8279.1990.tb00918.x

[5] RobinsonW: Ecological Correlations and the Behavior of Individuals. *American Sociological Review*.1950;15(3) : 10.2307/2087176 10.2307/2087176

# Response

**Published:** 2019-02-15

**Authors:** Pauline Frizelle

**Affiliations:** University College Cork, Ireland

3.1 Reliance on Thordardottir paper for predications a) narrative may not be representative of conversational performance b) MLU may not be a suitable measure of language complexity for older individuals

*a) The literature suggests that (in relation to typically developing children) narrative elicitation yields more syntactically complex language than conversation (Westerveld and Vidler, 2016). The advantage of narrative over conversational sampling has been repeatedly reported (see Leadholm & Miller, 1992; Wagner, Nettelbladt, Sahlén, & Nilholm, 2000; Southwood & Russell, 2004; Westerveld et al., 2004). We therefore valued this paper as one in which people with DS could show their potential in relation to producing complex syntactic constructions.*

*b) We agree that MLU does not reveal a comprehensive picture of syntactic knowledge as children grow older (see Frizelle et al, 2018). However it is considered a reliable measure until about 48 months of age (Klee & Fitzgerald, 1985; Rondal, Ghiotto, Bredart, & Bachelet, 1987; Blake, Quartaro, & Onorati, 1993) and the expressive language of many older individuals with DS is equivalent to that age. The main difficulty that we see in interpreting the Thordardottir paper is the lack of information on the participants’ cognitive ability.*

 3.2 Work from non-English speaking populations with Down syndrome that the authors could include

*Given that complex syntax unfolds differently in different languages and our participants were English speaking we deemed it the most appropriate paper on which we could base some predictions. We have now included information on the Christodoulou and Grohmann 2018 paper, although we note that the design of the their comprehension task is such that particpants with DS are likely to respond correctly without knowledge of complex structures.*

 3.3 Advantages to the use of TROG – perhaps providing concrete representations of response options at test makes aspects of a grammatic comprehension task easier, perhaps particularly for individuals with Down syndrome.

*It is possible that in relation to simple sentence constructions that providing a concrete representation of different response options may facilitate a best guess at the answer. However, our work in relation to complex sentences suggests that the presentation of three alternative responses (where thematic verb argument mapping is required) does not facilitate an accurate response but serves to increase the cognitive load for the child by operating as salient competitors rather than facilitators of an incomplete representation (see Frizelle, O’Neill & Bishop, 2017; Frizelle, Thompson, Duta & Bishop, 2018).*

 3.4 a) Our experience of working with participants with Down syndrome is that individuals often seem to struggle with the concept of ‘same vs. different’ and sometimes appear to have particular problems on tasks that require them to make such judgements. b) if aspects of the task are difficult for individuals with Down syndrome, for whatever reason, then it is possible that they might give up on the whole task to some extent.

*a) The first author worked clinically for a number of years with people with Down syndrome and agrees that some individuals do struggle with the concept of ‘same vs. different’ however our data does not suggest that this was a difficulty for our participants and this was reflected in their performance on the catch items and on their emerging performance on the intransitive subject relatives.*

*b) We saw no evidence of the participants with DS giving up. The reviewer is correct in that different sentence structures were interleaved with each other throughout the task. In addition there were 10 motivational items which we believe contributed considerably to the participants’ engagement with the task. Furthermore, because the individuals were seen over a number of sessions they did not have to sustain their attention for too long with each task.*

 3.5 To rule difficulties understanding the task, future work with this form of procedure might usefully include simple structures where one would expect individuals to perform well, or ‘acting out’ tasks that require the participant to recreate the meaning of the utterance with toy figures

*As the reviewers note elsewhere we included 10 catch items, the purpose of which was to reveal difficulties understanding what was required in the task and to reveal children showing a ‘yes’ bias. In addition, we included 6 practice items to assist with the participant’s understanding of the task. If children did not understand task requirements it is likely that they also had difficulty understanding complex syntax. In relation to recreating the meaning of the utterance with toy figures, we note previous literature in which this ‘act out’ method was criticized on the basis that it may underestimate children’s knowledge due to a competing acting bias i.e. children’s desire to play with the toys rather than follow the instructions they hear (see McDaniel & McKee, 1998).*

 3.6 Might it also be possible to examine the profile of performance of the subset of individuals with Down syndrome who appeared to clearly understand the TECS-E task? It might be worth including non-verbal MA as a covariate in the test of prediction 2.

 It may not be possible to carry out separate regression analyses for each group because of concerns about sample sizes and floor effects among the Down syndrome group, but it would be interesting to know whether verbal short-term memory and mental age are associated with performance on the TECS-E in just typically developing children.

*These are all interesting suggestions but we prefer to restrict our analyses to those we pre-registered. Of course the data are available for others to do so if they so wish.*

 3.7 Not quite accurate to suggest that the groups are matched

*We have rephrased this to explicitly state that there are no significant differences between the groups on a measure of non-verbal cognitive ability*

 Minor issues

*We have corrected the Table 1 labelling error*

*On page 9 we have changed the proability from <.035 to <.036*

*We have checked the reference order.*
